# Hybrid cell constructs consisting of bioprinted cell‐spheroids

**DOI:** 10.1002/btm2.10397

**Published:** 2022-08-31

**Authors:** WonJin Kim, GeunHyung Kim

**Affiliations:** ^1^ Department of Biomechatronic Engineering, College of Biotechnology and Bioengineering Sungkyunkwan University (SKKU) Suwon South Korea; ^2^ Biomedical Institute for Convergence at SKKU (BICS) Sungkyunkwan University Suwon South Korea

**Keywords:** cell printing, hASCs, spheroids, tissue engineering, vascularization

## Abstract

Bioprinted cell constructs have been investigated for regeneration of various tissues. However, poor cell–cell interactions have limited their utility. Although cell‐spheroids offer an alternative for efficient cell–cell interactions, they complicate bioprinting. Here, we introduce a new cell‐printing process, fabricating cell‐spheroids and cell‐loaded constructs together without preparation of cell‐spheroids in advance. Cells in mineral oil droplets self‐assembled to form cell‐spheroids due to the oil‐aqueous interaction, exhibiting similar biological functions to the conventionally prepared cell‐spheroids. By controlling printing parameters, spheroid diameter and location could be manipulated. To demonstrate the feasibility of this process, we fabricated hybrid cell constructs, consisting of endothelial cell‐spheroids and stem cells loaded decellularized extracellular matrix/β‐tricalcium phosphate struts for regenerating vascularized bone. The hybrid cell constructs exhibited strong angiogenic/osteogenic activities as a result of increased secretion of signaling molecules and synergistic crosstalk between the cells.

## 1 INTRODUCTION

Recently, cell printing has been used to fabricate a variety of complex tissue constructs, such as biomedical scaffolds and in‐vitro models used to evaluate therapeutic biocomponents, among others.^1^, ^2^, ^3^ This fabrication technique can provide biochemical/biophysical cues and even place cells in a desired region to achieve native tissue‐mimetic structures or patterns of multiple types of cells.^3^, ^4^, ^5^ Because cell printing allows encapsulation of cells into microscale hydrogel struts, such cell‐loaded structures have been widely used as tissue engineering substitutes for bone, skin, muscle, cardiac, and other tissues.^3^, ^4^, ^5^, ^6^, ^7^, ^8^ However, the homogeneously distributed cells in bioprinted three‐dimensional (3D) constructs often require extremely long times in culture to form the strong cell–cell attachments needed for a natural cellular microenvironment. For this reason, several researchers have been pursuing new innovative methods to induce strong cell–cell interactions within cell‐loaded constructs.^9^, ^10^, ^11^

**FIGURE 1:**
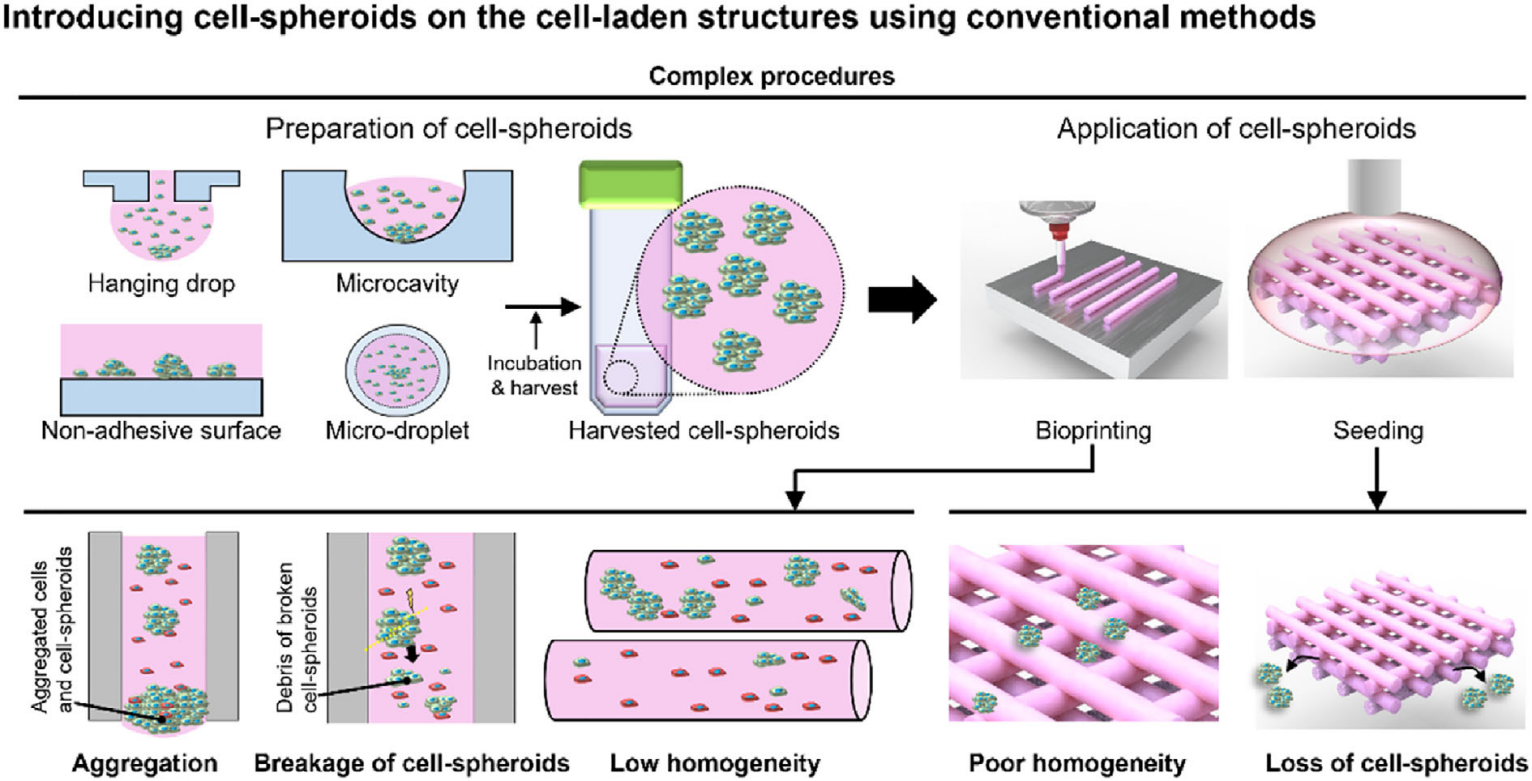
Application of conventionally prepared cell‐spheroids. Schematic drawings illustrating use and limitations of cell‐spheroids placed on cell‐loaded 3D scaffolds using conventional methods. 3D, three‐dimensional

Cell‐spheroids, which are 3D spherical aggregates of cells, have been used in tissue engineering applications because of their strong cell–cell/cell–ECM interactions that mimic those formed in cellular microenvironments in vivo.^12^, ^13^, ^14^ Cells in 3D spheroids have been reported to exhibit improved organ‐specific activities as a result of increased cell–cell communication via signaling factors, such as growth factors, chemokines, and cytokines, when compared with single cells in 2D culture.^14^, ^15^, ^16^ Due to the outstanding in‐vivo‐like 3D microenvironment, cell‐aggregates can be used not only as biomimetic tissue models, but also as models for various diseases for screening, diagnosis, treatment, and drug tests.^17^, ^18^, ^19^ Therefore, combining cell‐spheroids with bioprinting constitutes a synergistic approach that can overcome weak cell–cell interactions in cell‐loaded constructs fabricated by cell printing.^14^ To obtain spheroid‐based constructs, cell‐spheroids fabricated by conventional methods (e.g., hanging drop, microcavity, non‐adhesive surface) have been seeded into predesigned scaffolds by bioprinting a bioink that contains spheroids, as shown in Figure 1.^14^, ^20^, ^21^, ^22^, ^23^ However, a two‐step procedure can cause cell‐spheroid aggregation in the bioink, spheroid breakage resulting from high shear stresses near walls during extrusion through microscale printing nozzles, loss of spheroids, and low homogeneity of spheroids seeded into scaffolds (Figure 1). In this respect, efficient fabrication methods are needed to overcome these previous limitations of spheroid printing.^24^, ^25^

To address the issues, various methods have been proposed to hybridize the cell‐spheroids with hydrogels.^26^, ^27^, ^28^, ^29^ Williams et al.^26^ printed adipose stromal fraction cells encapsulated in alginate hydrogel into the CaCl_2_ solution. They successfully printed sphere‐shaped cell‐loaded alginate structures. Wu et al.^27^ also fabricated bone marrow stem cells‐loaded microspheres using gelatin methacryloyl (GeMA) hydrogel and a microfluidic system. By loading cells into the porous GelMA microspheres produced in advance, they obtained porous hydrogel‐based cell‐beads. While the cell‐beads have been produced with the hydrogels, several limitations for hybridizing the cell‐spheroids with 3D cell‐constructs need to be overcome.

To overcome the limitation, several advanced approaches including thermoplastic polymer frame works,^30^, ^31^ Kenzan,^32^ aspiration,^33^, ^34^ or dot‐printing^35^ techniques have been proposed (Table 1).^28^, ^36^, ^37^, ^38^ Mekhileri et al.^30^ printed thermoplastic polymer scaffold and inserted bioinks containing prepared cell‐spheroids. The Kenzan and aspiration methods produced complex spheroid blocks by accurately positioning the prepared spheroids using supporting needles and hydrogel matrix, respectively.^32^, ^33^, ^34^ These studies demonstrated the potential for efficient formation of cell‐spheroids‐based 3D constructs. However, multifaceted bioink preparations and complicated fabrication procedures need to be improved.

**TABLE 1:** Recent fabrication approaches for spheroids‐based 3D constructs

Method	Bioink	Spheroid formation	Supporting structure	Advantage	Limitation	Ref.
Bioprinting droplets of spheroid‐laden bioink	GelMA	Microgravity (PDMS microwell)	Non	‐ Precise positioning the droplets ‐ Application for on‐a‐chip platform ‐ Maintaining functions for long period	‐ Non‐homogeneous distribution of spheroid in droplet ‐ Requirement in prior‐preparation of spheroid	28
Microfluidic oil‐emulsion (singularization of spheroid)	GelMA/HepMA	V‐bottom plate	PEG/PBT frames	‐ Precise positioning the single spheroid ‐ Automated/scalable fabrication method	‐ Requirement in 3D frames ‐ Requirement in prior‐preparation of spheroid	30
Encapsulation in hydrogel	GelMA or Matrigel	Liquid‐overlay	ULA plate	‐ Available for drug screening system	‐ Requirement in prior‐preparation of spheroid ‐ Requirement in modification for obtaining complex 3D structure	36
Bioprinting	Alginate/PF127	Culturing on Matrigel	Non	‐ Efficient for obtaining multicellular 3D structure	‐ Possibility of spheroid deformation and non‐homogeneity	37
Kenzan method	Non	Ultra‐low attachment round‐bottom plate	Microneedle array	‐ Positioning the single spheroid ‐ Effective for obtaining scalable structure	‐ Requirement in supporting microneedles ‐ Requirement in prior‐preparation of spheroid	32
Bioprinting	Fibrin	Microgravity (PDMS microwell)	PEG/alginate hydrogel bath	‐ Reflecting 3D cartilage tissue environment ‐ Fast diffusion of nutrients ‐ Precise positioning the bioink	‐ Possibility of spheroid deformation ‐ Requirement in prior‐preparation of spheroid ‐ Requirement in supporting matrix	38
Aspiration‐assisted bioprinting	Non	Ultra‐low attachment round‐bottom plate	Hydrogel bath	‐ Precise positioning the single spheroid ‐ Efficient for obtaining complex scalable structure	‐ Requirement in sacrificial hydrogel bath ‐ Requirement in prior‐preparation of spheroid	33, 34
Bio‐dot printing	HA/gelatin/alginate	In‐situ formation	PCL frames/supporting matrix	‐ Precise control of spheroid size and position ‐ Efficient for multicellular micro‐patterning ‐ Cells loaded in the supporting matrix	‐ Requirement in frames and supporting matrix	35

Abbreviations: 3D, three‐dimensional; GelMA, gelatin methacryloyl; HA, hyaluronic acid; HeMA, methacrylated heparin; PEG/PBT, poly(ethylene glycol)‐poly(butylene terephthalate) block copolymers; PF127, pluronic F‐127; ULA plate, ultra‐low adhesive plate.

In this study, a new cell‐printing system incorporating in‐situ spheroid formation has been developed to fabricate 3D bioengineered constructs containing multiple types of cells as well as precise patterns of the printed cell‐spheroids. A one‐step hybrid printing process was developed to simultaneously fabricate both cell‐spheroids with similar biological functions to the conventional cell‐spheroids, and cell‐printed constructs without preparation of cell‐spheroids in advance. Cell‐spheroids were obtained by printing cells in droplets of mineral oil, and the spheroids were designed to influence biofunctional characteristics of surrounding cells loaded into supporting struts. Cell‐spheroid size and location were easily manipulated by controlling various printing parameters.

To show the effect of these hybrid constructs, endothelial cells were used for cell‐spheroids, and human adipose stem cells (hASCs)‐loaded bioink was used to fabricate the cell‐laden struts for regenerating bone tissue. In vitro cellular activities of these constructs were significantly improved over those of conventional cell constructs printed using a mixture of the endothelial cells and hASCs. These differences were a result of reinforced synergistic crosstalk between the endothelial spheroids and hASCs, which accelerated angiogenesis and osteogenesis in vitro.

## 2 EXPERIMENTAL SECTION

### 2.1 Cells and bioinks

In the present study, human adipose‐derived stem cells (hASCs; Lonza, USA) and human umbilical vein endothelial cells (HUVECs; Lonza, USA) were used to formulate bioinks. Before preparing the bioinks, the cells were cultured in cell culture plates with different culture media at 37°C and 5% CO_2_. Dulbecco's Modified Eagle's Medium‐low‐glucose (DMEM‐L; Sigma‐Aldrich, USA)‐based GM, containing 10% fetal bovine serum (BioWest, USA) and 1% penicillin–streptomycin (PS; Thermo‐Fisher Scientific, USA), were used for hASCs. In the case of HUVECs, an EGM™‐2 endothelial SingleQuots™ kit supplemented with EBM™‐2 (Lonza, USA) containing 1% PS (EBM) was used. GM and EBM were changed every 2 days. To formulate bioinks in which spheroids formed, the hASCs and HUVECs (2.0 × 10^7^ cells/ml) were mixed with mineral oil (Sigma‐Aldrich, USA).

Before preparing bone‐specific bioinks, bone tissues were isolated from the lower limbs of Yorkshire pigs (females, 10–15 months old) and demineralized/decellularized in accordance with protocols described in previous paper.^39^ The isolated bone tissue was crushed to obtain bone powder after rinsing several times with Dulbecco's phosphate‐buffered saline (DPBS; Biowest, USA) and deionized water (DW). The bone powder was demineralized by treatment with 0.5 M HCl (Sigma‐Aldrich, USA) for 5 h with continuous stirring at room temperature. Following removal of the remaining solution, the treated powder was washed at least three times with DW. Then, the remaining lipid in the powder was removed by treatment with lipid removal solution containing chloroform (Sigma‐Aldrich, USA) and methanol (Sigma‐Aldrich, USA) at a ratio of 1:1. After 1 h of treatment, the solution was rinsed with methanol and five times with DW and then freeze‐dried in a freeze‐drier (SFDSM06; Samwon, South Korea). The demineralized bone matrix (DBM) was stored at −80°C before decellularization.

The lyophilized DBM was incubated in trypsin (0.05%)–ethylenediamine tetra‐acetic acid (0.02%; EDTA) (TE; Sigma‐Aldrich, USA) solution for 2 h at 37°C. After removing the TE solution, DBM powder was washed thrice with DW and soaked in 70% ethanol for 1 day. Then the powder was thrice washed with DW and lyophilized and stored at −80°C before solubilization. The freeze‐dried decellularized tissue was digested in pepsin solution (0.1% w/v in 0.5 M acetic acid; Sigma‐Aldrich, USA) at room temperature for 2 days, and precipitation was performed by adding sodium chloride (Sigma‐Aldrich, USA). Then, the solution was dialyzed (1000 kDa molecular cut‐off; Spectrum Chemical Manufacturing, USA) at 4°C for 3 days. The dialyzed soluble bone decellularized extracellular matrix (dECM) were lyophilized and stored at −80°C.

To obtain the cell‐loaded composite bioink, the prepared dECM sponge and β‐TCP powder (Sigma‐Aldrich, USA) were mixed under the previously reported condition.^39^, ^40^, ^41^, ^42^ Briefly, dECM was dissolved in 0.1 M acetic acid solution and mixed with 10 × DMEM (Sigma‐Aldrich, USA) at a ratio of 1:1 to neutralize the dECM hydrogel. The neutralized dECM (0.05 g/ml) hydrogel was mixed with β‐TCP (0.2 g/ml) using three‐way stopcock (Hyupsung Medical Co., Ltd., South Korea) and syringes (Korea Vaccine Co., Ltd., South Korea). Then, the cells were mixed with the dECM/β‐TCP composite under following conditions: (1) 1.2 × 10^7^ cells/ml of hASCs, (2) 2.0 × 10^7^ cells/ml of hASCs, and (3) 0.8 × 10^7^ cells/ml of HUVECs and 1.2 × 10^7^ cells/ml of hASCs.

### 2.2 One‐step spheroid printing

To fabricate the spheroid‐based cell‐printed construct, a 3D bioprinting system (DTR3‐2210 T‐SG; DASA Robot, South Korea), consisting of a pneumatic pressure dispenser (AD‐3000C; Ugin‐tech, South Korea) and a dual‐barrel, was used to print cell‐loaded bioink and mineral oil at controlled (barrel: 20°C; plate: 37°C). After printing hASC (1.2 × 10^7^ cells/ml)‐laden dECM (5 wt%) bioink struts at a speed of 5 mm/s using 25G nozzle (diameter = 250 μm) and pneumatic pressure (80 kPa), HUVEC (2.0 × 10^7^ cells/ml)‐loaded mineral oil droplets were deposited onto the struts using 30G nozzle (diameter = 150 μm). The flow rate of the mineral oil and movement speed of the nozzle could be manipulated to produce a range of spheroid diameters.

To obtain diverse patterns of spheroids, HUVEC (2.0 × 10^7^ cells/ml)‐loaded mineral oil droplets were deposited onto the dECM struts (nozzle diameter = 150 μm, oil flow rate = 3.8 μl/min, and movement speed = 5 mm/s) after locating hASC (2.0 × 10^7^ cells/ml)‐loaded mineral oil droplets (nozzle diameter = 150 μm, oil flow rate = 1.1 μl/min, and movement speed = 5 mm/s).

In the case of the spheroid‐based cell construct for bone tissue (EXP construct), hASC (1.2 × 10^7^ cells/ml)‐loaded dECM/β‐TCP (ratio = 2:8) composite bioink and HUVEC (2.0 × 10^7^ cells/ml)‐loaded mineral oil were used. Following fabrication of the hASC‐loaded dECM/β‐TCP structure (nozzle diameter = 250 μm, pneumatic pressure = 90 kPa, and movement speed = 5 mm/s), HUVEC‐loaded oil droplets were deposited onto the struts (nozzle diameter = 150 μm, volume flow rate = 3.8 μl/min, and movement speed = 5 mm/s). The procedures were repeated to obtain a 3D cell construct. The printed constructs were cultured in GM at 37°C and 5% CO_2_. The GM was changed every 2 days.

As controls for the EXP construct, two types of bioinks were printed under the same fabrication conditions without depositing HUVEC‐loaded mineral oil: hASC (2.0 × 10^7^ cells/ml)‐loaded (CON‐1) and HUVEC (0.8 × 10^7^ cells/ml)/hASC (1.2 × 10^7^ cells/ml)‐loaded (CON‐2) dECM/β‐TCP composite bioinks. The number and ratio of cells were adjusted to be the same as those of the EXP construct, estimated with the MTT assay.

### 2.3 Deposition of cell‐loaded mineral oil droplets into collagen hydrogels

To observe formation of cell‐spheroids in mineral oil, HUVEC‐loaded mineral oil and collagen hydrogel were used. Briefly, mineral oil, alginate (4 wt%), or collagen (5 wt%) droplets (0.1 μl) loaded with HUVECs were added to collagen hydrogel (5 wt%) in 96‐well plates using a micro pipette (Gilson Inc., France). The collagen hydrogel containing cell‐loaded droplets was cultured in GM at 37°C and 5% CO_2_. The GM was changed every 2 days.

### 2.4 Preparation of conventional HUVEC‐spheroids

Conventional HUVEC‐spheroids (~200 μm diameter) were prepared using non‐adherent agarose molds in accordance with the manufacturer's protocols to confirm the efficacy of generated one‐step spheroid printing (One‐SP)‐spheroids.^43^ Briefly, the agarose molds were prepared by casting 2% agarose (in DPBS; Invitrogen, USA) into a 3D Petri dish (Sigma‐Aldrich, USA). Then, the HUVEC suspension (190 μl; 1.4 × 10^6^ cells/ml) was seeded into the agarose mold and cultured with GM.

### 2.5 Cellular responses of hASCs to HUVEC‐spheroids

To evaluate the osteogenic responses of stem cells to endothelial cells and spheroids, hASCs (1 × 10^5^ cells/cm^2^) were seeded onto 6‐well tissue culture plates. The One‐SP‐spheroids‐based dECM construct (containing 56 spheroids) was placed onto the top level of transwell inserts (SPL Life Science, USA). As controls, 2D cultured isolated HUVECs and conventionally prepared HUVEC‐spheroids (C‐Spheroids) were positioned on the top level in a dECM construct. The GM was changed every 2 days, and the cells were cultured at 37°C and 5% CO_2_.

### 2.6 Characterization of cell constructs

The 3D printed constructs were visualized using an optical microscope (BX FM‐32; Olympus, Japan) with a digital camera and a scanning electron microscope (SEM) (SNE‐3000M; SEC Inc., South Korea). Before observing surface morphology of the constructs using SEM, constructs were treated with 10% neutral buffered formalin (NBF; Sigma‐Aldrich), dehydrated with an ethanol series (50%, 60%, 70%, 80%, 90%, and 100%), and lyophilized.

The mechanical properties of the bioprinted constructs (6 × 6 × 3 mm^3^) were estimated using a SurTA universal testing machine (Chemilab, South Korea) in compressive testing mode (compression rate: 0.1 mm/s). Following measurement of stress–strain curves, the compressive moduli were calculated over the linear regions of the curves. All values are presented as means ± SDs (*n* = 4).

To measure the content of bioceramics in the fabricated constructs, thermogravimetric analysis was performed under a nitrogen atmosphere using a thermogravimetric analyzer (TGA‐2050; TA‐Instruments, USA). The freeze‐dried scaffolds were heated from 30°C to 800°C (typical sample mass: 10 mg; ramp rate: 20°C/min).

### 2.7 In‐vitro cellular responses

To estimate numbers and proliferation of cells, the MTT assay was performed using the Cell Proliferation Kit I (Boehringer, Mannheim, Germany). After thrice rinsing samples with DPBS, they were incubated in 3‐(4,5‐dimethylthiazol‐2‐yl)‐2, 5‐diphenyltetrazolium bromide (MTT) solution for 4 h at 37°C. Metabolically active cells produced purple formazan crystals. The insoluble crystal was dissolved by adding solubilization solution containing sodium dodecyl sulfate. The optical density (OD) of colored solutions was measured using a microplate reader (EpochTM; BioTek, South Korea) at 570 nm. The cell number for measured OD was calculated using the following equation:

Cell number=161,459×OD−13,123

obtained from a standard curve of the ODs of known cell numbers. All values are expressed as means ± SDs (*n* = 4).

To observe viable hASCs and HUVECs, cells in the 3D constructs were visualized by staining with calcein AM (0.15 mM; Invitrogen, USA) and ethidium homodimer‐1 (2 mM; Invitrogen, USA). The stained live (green) and dead (red) cells were visualized using a laser‐scanning confocal microscope (LSM 700; Carl Zeiss, Germany), and the numbers of live and dead cells were counted to estimate cell viability using ImageJ software (National Institutes of Health, USA). All values are expressed as means ± SDs (*n* = 4).

To separately visualize the printed hASCs and HUVECs, the cells were prestained using Cell‐Tracker™ (Molecular probes, USA) according to the manufacturer's protocol before formulating the bioinks. After cells were harvested, they were incubated in prewarmed staining solution at 37°C for 30 min and then removed from the staining solution. The stained hASCs (red) and HUVECs (green) were tracked after fabrication using the Zeiss confocal microscope.

By staining hASCs and HUVECs with diamondino‐2‐phenylinodole (DAPI) (Invitrogen, USA) and Alexa Fluor 594‐conjugated phalloidin (Invitrogen, USA), nuclei and F‐actin were observed. Briefly, the cells were fixed (30 min) and permeabilized (10 min) with 10% NBF and 0.1% Triton X‐100 (Sigma‐Aldrich, USA), respectively, at 37°C. Then, the specimens were incubated in DAPI (1:100 in DPBS)/phalloidin (1:100 in DPBS) solution (for 1 h) at 37°C, and the stained nuclei (blue) and F‐actin (red) were visualized under the Zeiss confocal microscope. The diameter of spheroids and the area and length of endothelial sprouts were measured with ImageJ software. All values were expressed as means ± SDs (*n* = 4).

The total protein content of the hASCs cultured on the six‐well culture plates was examined at 14 and 28 days using the Pierce™ BCA (bicinchoninic acid) protein assay kit (Thermo‐Fisher Scientific, USA). The cultured cells were thrice rinsed with DPBS and treated with 0.1% Triton X‐100. Then, BCA working solution (200 μl) was added to the lysate (25 μl) and incubated for 30 min at 37°C. The OD was measured at 562 nm using the microplate reader, and known standards were used to determine protein concentration (*n* = 4).

To evaluate osteogenic differentiation of hASCs cultured on 2D plates, cells were stained for alkaline phosphatase (ALP) and calcium. Alizarin red S (ARS) was used to stain for calcium, and nitro blue tetrazolium chloride (NBT; Sigma‐Aldrich, USA)/5‐bromo‐4‐chloro‐3‐indolyl‐phosphate, 4‐toluidine salt (BCIP; Sigma‐Aldrich, USA) solution was used to perform ALP staining at 14 days in culture. The cells were osmotically balanced using AP buffer consisting of Tris‐Cl (100 mM, pH 9.5), NaCl (100 mM), and MgCl_2_ (10 mM), after rinsing twice with DPBS. Then, the balanced hASCs were stained using NBT/BCIP solution for 30 min, followed by termination of the reaction with EDTA (20 mM in DPBS). To visualize the stained cells, the Olympus optical microscope was used.

After 14 and 28 days, hASCs were fixed with 70% ethanol (Sigma‐Aldrich, USA) for 1 h at 4°C, and cells were stained with 40 mM ARS (pH 4.2; Sigma‐Aldrich, USA) for 1 h at room temperature. Stained cells were thrice washed with DW and observed using the optical microscope. To estimate the calcium level, the cells were destained by treatment with 10% cetylpyridinium chloride (Sigma‐Aldrich, USA) dissolved in sodium phosphate buffer (10 mM, pH 7.0) for 30 min at room temperature. The absorbance of the cells and known standards were measured using the microplate reader at 562 nm and normalized to total protein. All values are expressed as means ± SDs (*n* = 4).

### 2.8 Immunofluorescence

To evaluate differentiation of stem cells and endothelial cells, they were fixed with 10% NBF for 1 h, blocked with 2% bovine serum albumin (BSA; Sigma‐Aldrich) for 2 h, and permeabilized with 2% Triton X‐100 for 2 h. The samples were incubated with an anti‐mouse osteopontin (OPN) primary antibody (5 μg/ml; Invitrogen, USA) and an anti‐rabbit CD31 primary antibody (5 μg/ml; Invitrogen, USA) overnight at 4°C. The primary antibody‐treated samples were stained with Alexa Fluor 488‐conjugated anti‐mouse secondary antibody (1:50 in DPBS; Invitrogen, USA) or Alexa Fluor 594‐conjugated anti‐rabbit secondary antibody (1:50 in DPBS; Invitrogen, USA) for 1 h, and counterstained with DAPI (5 μM in DPBS). The confocal microscope was used to visualize the stained cells. OPN‐ and CD31‐positive areas were quantified using ImageJ software. All values are expressed as means ± SD (*n* = 4).

### 2.9 Quantitative reverse transcription polymerase chain reaction

To evaluate gene expression in cultured hASCs and HUVECs, quantitative reverse transcription polymerase chain reaction (RT‐qPCR) was performed on the cells using the 2^−∆∆CT^ method. Total RNA was isolated from the cultured cells by treating them with TRIzol reagent (Sigma‐Aldrich, USA). The purity of the isolated RNA was evaluated using a FLX800T spectrophotometer (Biotek, USA) (acceptable purity: 1.8 < OD260/OD280 < 2.0). Then, a reverse transcription process was performed to synthesize cDNA using the RNase‐free DNase‐treated total RNA and qPCR RT Master Mix (ReverTra Ace™; Toyobo Co., Ltd., Japan). The synthesized cDNA was used to perform RT‐qPCR by measuring threshold cycle (CT) values using a real‐time PCR system (StepOnePlus; Applied Biosystems, USA) and qPCR mix (Thunderbird® SYBER®; Toyobo Co., Ltd., Japan). Measured levels of the glyceraldehyde 3‐phosphate dehydrogenase (*Gapdh*) gene were used as a housekeeping gene to normalize the expressed gene levels. All values are reported as means ± SDs (*n* = 4). The gene‐specific primers are shown in Table S1.

### 2.10 Statistical analysis

Student's *t*‐test was used (two groups), and a single‐factor analysis of variance (ANOVA) with Tukey's Honest Significant Difference (HSD) post‐hoc test (three or more groups) was performed to evaluate statistical analyses using SPSS software (SPSS, Inc., USA). Values of *p < 0.05, **p < 0.01, and ***p < 0.001 were considered statistically significant.

## 3 RESULTS AND DISCUSSION

### 3.1 In‐situ formation of cell‐spheroids using mineral oil and 3D printing

Cell‐spheroids are usually prepared by seeding and culturing cells onto a non‐adhesive surface where they self‐assemble into spherical clusters.^20^ However, there are several limitations to combining conventionally prepared spheroids with 3D printing for control of spheroid size and position within spheroid‐based constructs using bioink mixed with separately fabricated spheroids and biocompatible hydrogels (Figure 1). To address the limitations, various researchers have proposed efficient approaches to obtain cell‐spheroid‐based scaffolds (Table 1). However, preparation periods to form cell‐speroids^28^, ^30^, ^31^, ^32^, ^33^, ^34^, ^35^, ^36^, ^37^ or additional supporting frames/hydrogel bath^30^, ^31^, ^32^, ^33^, ^34^, ^35^, ^36^ are required to successfully achieve the spheroid‐laden scaffolds. Hence, we have focused on the fabrication of the hybrid cell constructs consisting of cell‐spheroids and cell‐loaded constructs together via a simultaneous bioprinting system.

**FIGURE 2:**
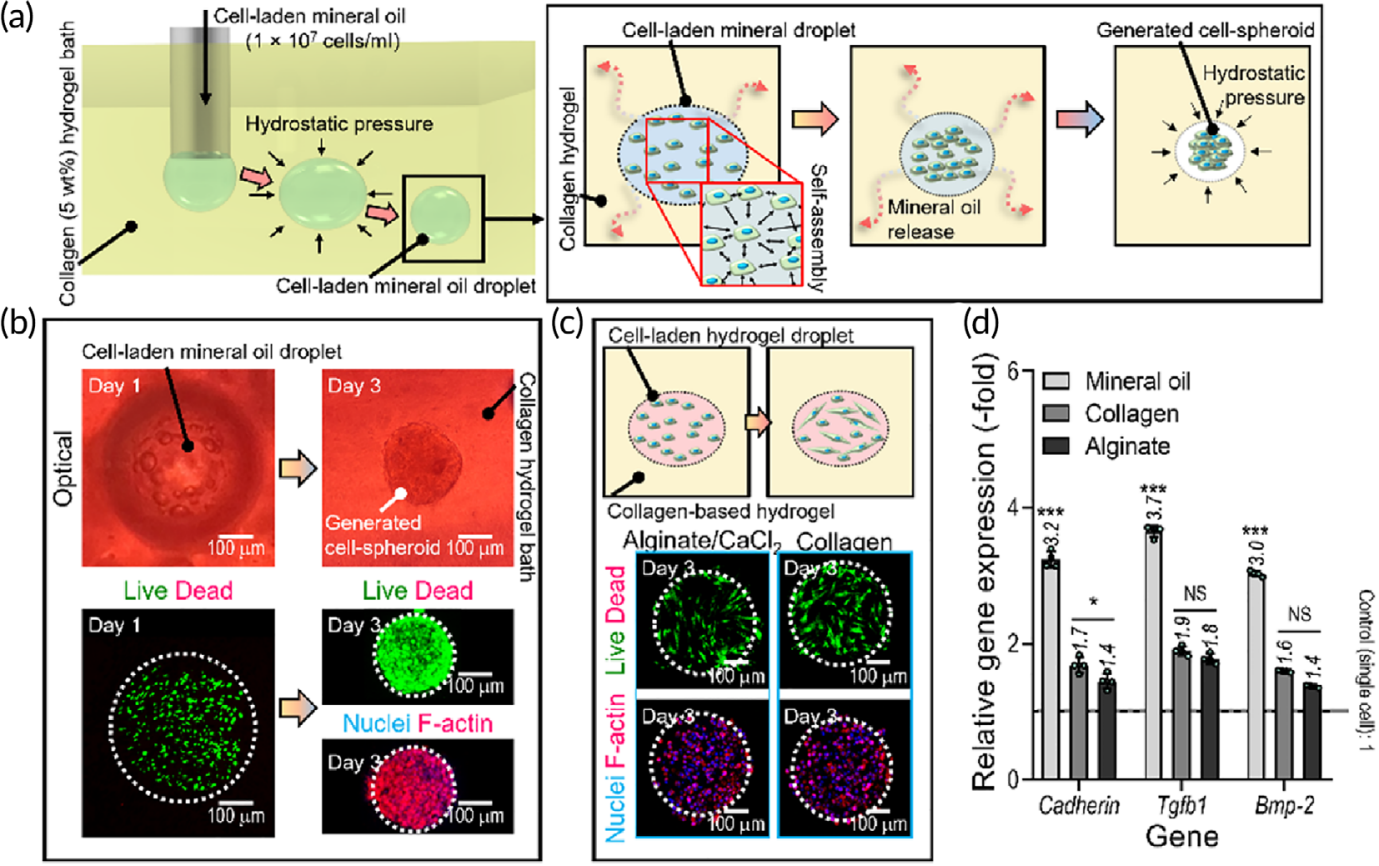
Fabrication of cell‐spheroids using cell‐loaded mineral oil. (a) Schematic drawings illustrating the deposition of a cell‐loaded mineral oil droplet into a collagen‐based hydrogel leading to the formation of a cell‐spheroid. (b) Optical and live (green)/dead (red) and nuclei (blue)/F‐actin (red) images of cells loaded into a droplet of mineral oil at 1 and 3 days. (c) Schematic drawings and fluorescence images (live/dead and nuclei/F‐actin) at 3 days of cells loaded into droplets of alginate and collagen hydrogels within a collagen hydrogel. (d) Expression of *Ve‐cadherin*, *Tgfb1*, and *Bmp‐2* genes at 7 days in cells loaded into mineral oil, collagen, and alginate droplets (*n* = 4). *p < 0.050, ***p < 0.001, one‐way ANOVA with Tukey's HSD post‐hoc test. ANOVA, analysis of variance; Tukey's HSD, Tukey's Honest Significant Difference

To achieve a unique construct based on cell‐spheroids, we developed a method to fabricate the spheroids by using mineral oil droplets loaded with cells. To demonstrate the feasibility of this method of spheroid formation, a cell‐loaded mineral oil droplet was placed in collagen solution (5 wt%), as shown in Figure 2a. In the present work, we used human umbilical vein endothelial cells (HUVECs) to fabricate cell‐spheroids. A collagen solution can naturally induce spherical formation of the cell‐loaded mineral oil microdroplets by minimizing surface forces at the aqueous–oil interface. Furthermore, during culture of the cell‐loaded oil droplet in the collagen solution, the mineral oil inhibited the cellular attachment to the collagen phase, thus forcing the cells together into an aggregate‐forming cell‐spheroid,^44^ as shown in the schematic drawings of Figure 2a. The oil components have been released from the collagen solution. This process was monitored by optical imaging (live (green)/dead (red) cells, nuclei (blue), and F‐actin (red); Figure 2b). As can be seen in the images, the cells in the mineral oil droplet formed spherical aggregates over a period of 3 days in growth medium (GM). However, when cells in hydrogels (4 wt% alginate or 5 wt% collagen), rather than mineral oil, were placed in the same collagen solution, the encapsulated cells did not aggregate, and they adhered and even proliferated in the hydrogels (Figure 2c). These differences can be explained by the cells easily adhering to the bioink hydrogel matrix rather than aggregating and adhering to each other at the interface between the droplet and collagen solution. To quantitatively assess the effects of cell aggregation, RT‐PCR was used to measure expression of proteins involved in intercellular communication in the three cell‐loaded groups (cell‐loaded mineral oil, alginate, and collagen; Figure 2d). It was found that *Ve‐cadherin*, *Tgfb‐1*, and *Bmp‐2* genes were significantly upregulated when the cells were aggregated in mineral oil droplets after 7 days of culture, much more so than for cells in alginate and collagen hydrogels.

In addition, to simply observe the effect of the mineral oil on the cell viability and spheroid formation, we cultured HUVECs in growth medium with mineral oil and showed that the cell viability and maintenance of cell‐spheroids were not affected by the mineral oil (Figure S1).

### 3.2 Combined in‐situ cell‐spheroid formation and cell printing

**FIGURE 3:**
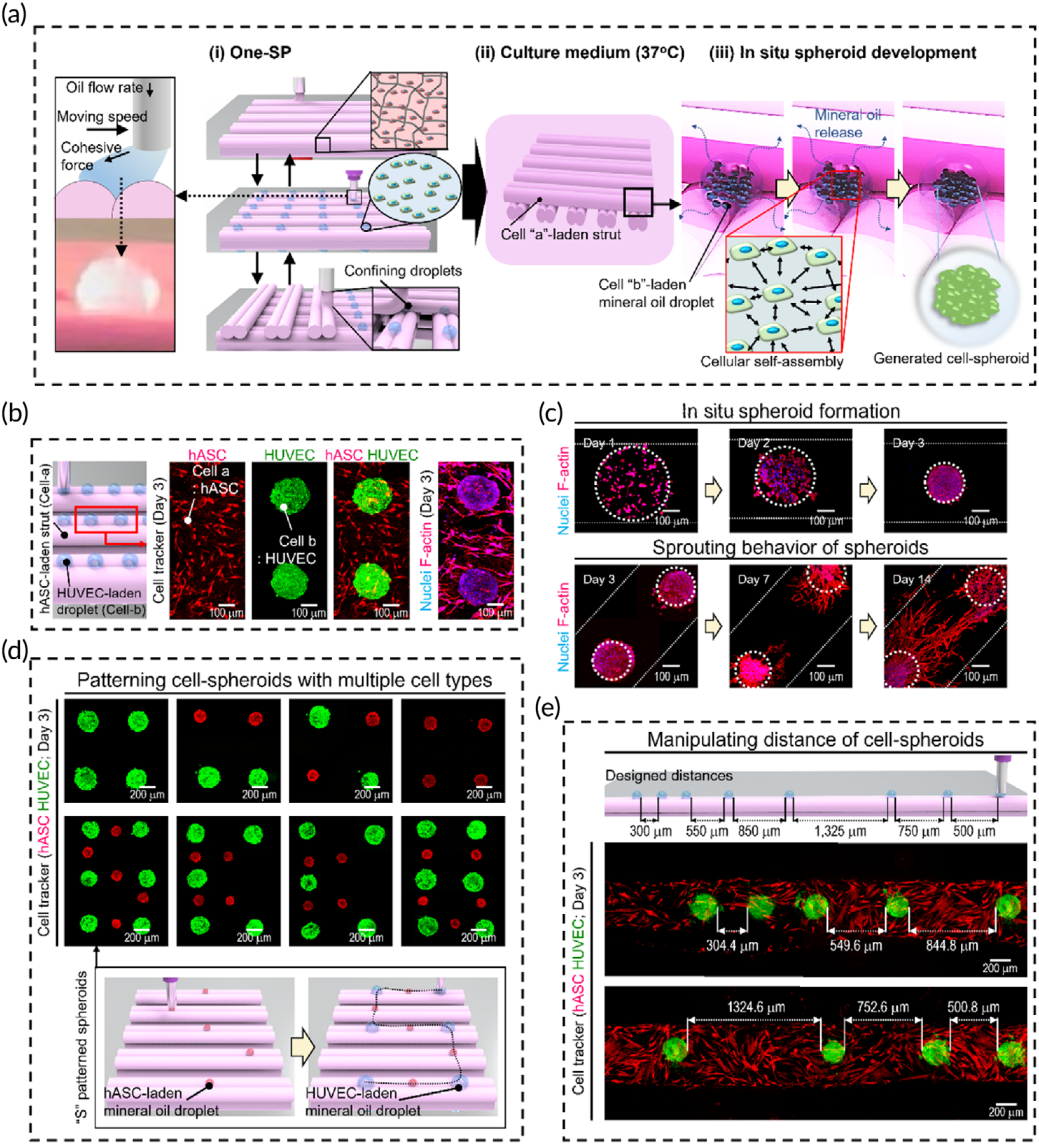
Fabrication of spheroid‐based cell constructs via One‐SP. (a) Schematic diagrams of the cell‐printing system illustrating (i) One‐SP, (ii) incubation of the fabricated construct containing individual cells and spheroids (cell ‘a’‐loaded struts and cell ‘b’‐loaded spheroids, respectively) in culture medium, and (iii) spheroid formation within the struts. (b) Schematic drawings and fluorescence images for hASCs (red) loaded into the printed struts and HUVECs (green) in spheroids, and nuclei/F‐actin images of the spheroid‐based cell construct at 3 days of cell culture. (c) Nuclei/F‐actin images demonstrating formation of spheroids at 1, 2, and 3 days in culture, and sprouting of two HUVEC‐spheroids at 3, 7, and 14 days. Schematic diagrams and cell tracker images (at 3 days) of (d) hASC (red)‐ and HUVEC (green)‐spheroids in various patterns, and (e) HUVEC (green)‐spheroids separated by various distances (300, 550, 850, 1325, 750, 500 μm) within the hASC (red)‐loaded struts. hASCs, human adipose stem cells; HUVECs, human umbilical vein endothelial cells; One‐SP, one‐step spheroid printing

To achieve in‐situ cell‐spheroid formation using the cell‐loaded mineral oil droplets, a cell‐printing system was used for effective spheroid formation within a 3D meshwork of printed cylindrical struts, as shown in Figure 3a. We call this process one‐step spheroid printing.

As shown schematically in Figure 3a(i), generation of cell‐spheroids occurred in the groove between a pair of printed parallel struts loaded with one type of cell (‘cell‐a’), onto which a droplet of mineral oil containing a second type of cell (‘cell‐b’) was printed. Then, another pair of struts was printed on top of the cell‐loaded mineral oil droplets to hold them in place. These steps were repeated several times to produce a 3D mesh structure. The fabricated constructs were then cultured in GM at 37°C and 5% CO_2_ (Figure 3a[ii]). As oil continuously diffused out from the droplet during culture, the cells aggregated into spheroids in the droplets between the struts without adhering to them because the oil–aqueous interface inhibited the cellular attachment (Figure 3a[iii]).

To demonstrate the feasibility of this process, we used hASCs as ‘cell‐a’ encapsulated in struts composed of dECM bioink derived from porcine bone. HUVECs acted as ‘cell‐b’ within the mineral oil used to fabricate the cell‐spheroids. As there was no released mineral oil after 5 days in GM (Figure S2), we carefully considered that the oil component has been sufficiently removed from the constructs. After 3 days in culture, cells, F‐actin, and cell nuclei in the fluorescently labeled HUVEC‐spheroids and hASC‐loaded struts were observed in fluorescence images (Figure 3b). The HUVECs were observed to aggregate into cell‐spheroids, and the hASCs were homogeneously dispersed throughout the struts. In particular, the nuclei/F‐actin images in Figure 3c showed that the HUVECs were initially in compact aggregates, but over a period of days migrated out of the spheroids and into the struts to form endothelial sprouts that eventually connected adjacent spheroids. These observations demonstrated that the One‐SP method successfully induced cell‐spheroids within the printed cell‐loaded construct without separate preparation of spheroids or sacrificial components.

Figure 3d shows images of various patterns of spheroids containing fluorescently labeled cells (hASC [red] or HUVEC [green]) in mineral oil. Different flow rates were used for printing the two types of cell‐loaded mineral oil (hASC: 1.1 μl/min; HUVEC: 3.8 μl/min) at a constant speed of nozzle movement (5 mm/s). The diameters of the spheroids were 141.8 μm for hASCs and 206.1 μm for HUVECs. Furthermore, the distance between the cell‐spheroids could be controlled during printing (Figure 3e).

### 3.3 Procedure for stable in‐situ spheroid formation in printed constructs

In the One‐SP method, stable cell‐spheroids with controllable diameters could be formed in the groove between paired dECM struts via manipulation of the flow rate of the cell‐loaded mineral oil and the speed of the nozzle. As the maximum size of deposited mineral oil droplets and formed cell‐spheroids could be affected by the strut size (Figure S3), we set the size of paired struts below 400 μm for the supplying nutrients and oxygen efficiently to the cells loaded in the struts.^45^

**FIGURE 4:**
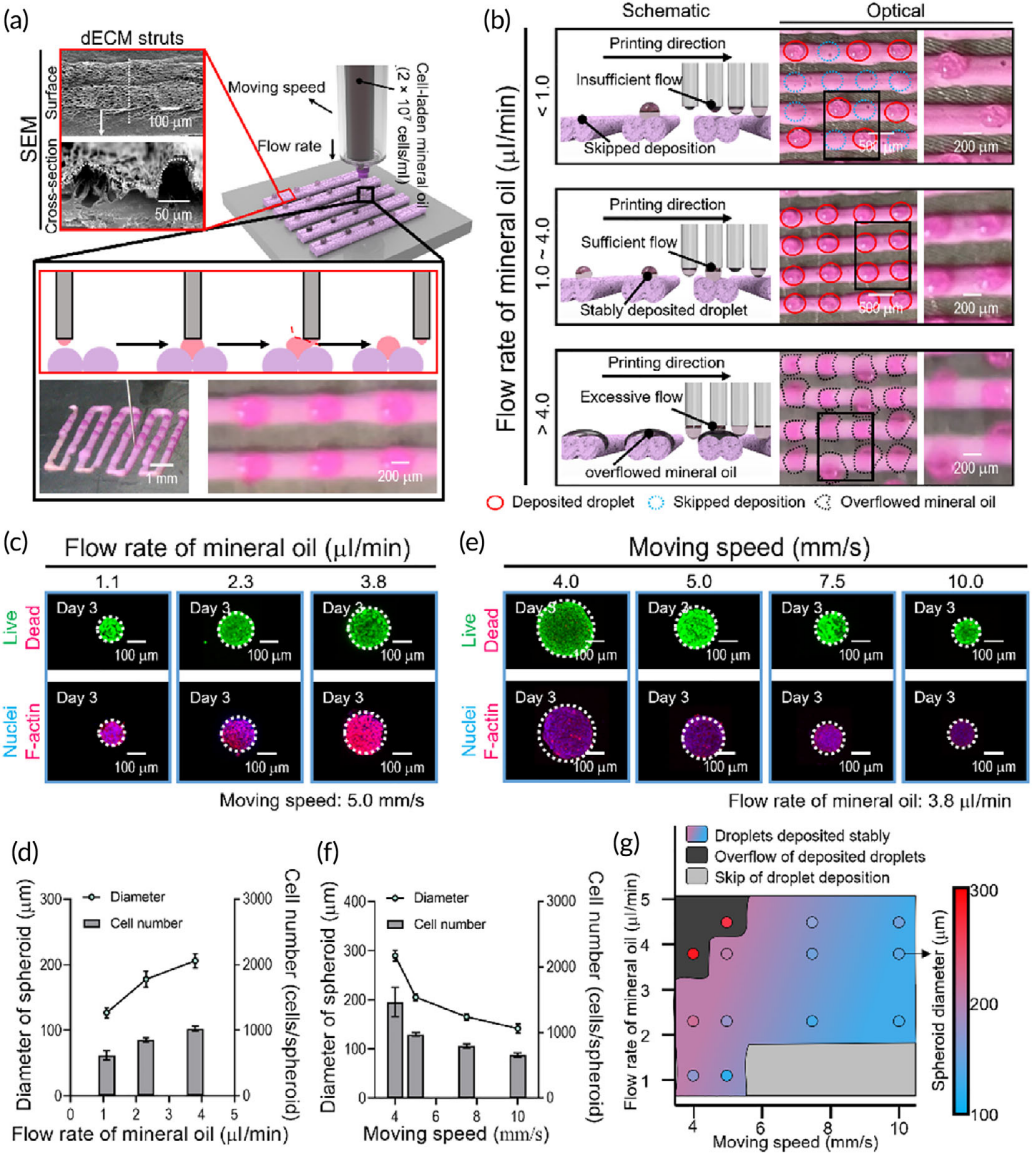
Selection of optimal printing parameters for stable spheroid formation using the One‐SP method. (a) Schematic diagrams illustrating placement of mineral oil droplets that contain cells. (b) Schematic diagrams and optical images illustrating the effects of volume flow rate on the formation of droplets. (c) Live/dead and nuclei/F‐actin images of cell‐spheroids at 3 days after having been printed at three different oil flow rates (1.1, 2.3, 3.8 μl/min). (d) Diameter (*n* = 20) and cell number (*n* = 4) of spheroids resulting from printing at the three flow rates. (e) Live/dead and nuclei/F‐actin images and (f) calculated diameter (*n* = 20) and cell number (*n* = 4) of spheroids printed at four speeds of nozzle movement (4.0, 5.0, 7.5, and 10.0 mm/s) at 3 days. (g) A process diagram for spheroid formation (including diameter) via One‐SP using various oil flow rates and nozzle movement speeds. One‐SP, one‐step spheroid printing

The effects of flow rate and nozzle movement speed on the formation of cell‐spheroids are shown in the SEM image of Figure 4a. The effect of flow rate on formation of a stable droplet on the dECM surface (cell density: 2 × 10^7^ cells/ml) is shown in Figure 4b. At a fixed nozzle movement speed of 5 mm/s, overflow of the mineral oil could occur when flow rate was relatively high (>4 μl/min), whereas a low flow rate (<1 μl/min) was insufficient to produce an oil droplet at every location. Based on these results, we used flow rates of about 1–4 μl/min.

After encapsulating cell‐loaded mineral oil droplets within perpendicularly arranged dECM struts, they were analyzed by live/dead cell and nuclei/F‐actin imaging after culturing for 3 days (Figure 4c). As shown in the images, the cells in the mineral oil droplet were aggregated, and most survived, and cell‐spheroid diameter continuously increased with an increase in flow rate, indicating that the number of cells in the spheroids could be easily controlled (Figure 4d).

In addition, the effect of nozzle movement speed was observed at a fixed mineral oil flow rate (3.8 μl/min). As expected, faster movement resulted in significantly reduced spheroid diameters (Figure 4e). The measured spheroid diameter and number of cells in each are shown in Figure 4f. Because the dependence of spheroid formation on flow rate and nozzle movement speed was complex, a process diagram consisting of three regions (stable spheroid formation, overflow of the mineral oil, and insufficient droplet size) and indicating spheroid diameter (after culturing for 3 days) at a fixed geometry of the dECM struts and cell density of 2 × 10^7^ cells/ml is shown in Figure 4g.

### 3.4 Cellular activities of the printed HUVEC‐spheroids

The microenvironment within cell‐spheroids resembles that in vivo in terms of robust cell–cell and cell–matrix interactions.^20^, ^21^ In particular, cells exhibit greatly upregulated tissue‐specific gene expression and secrete various intercellular signaling molecules, including cytokines, chemokines, and growth factors, at higher levels than do cells in 2D monoculture.^14^, ^15^, ^16^, ^46^, ^47^

In this section, HUVEC‐spheroids fabricated by the One‐SP method were assessed by comparing them with single cells in 2D culture and cell‐spheroids prepared using a conventional microwell process, as shown in Figure S4. Conventional cell‐spheroids (C‐spheroids) were fabricated using an agarose mold (seeding cell density, 1.4 × 10^8^ cells/ml), and optical images of the aggregated cells are shown in Figure S4.

**FIGURE 5:**
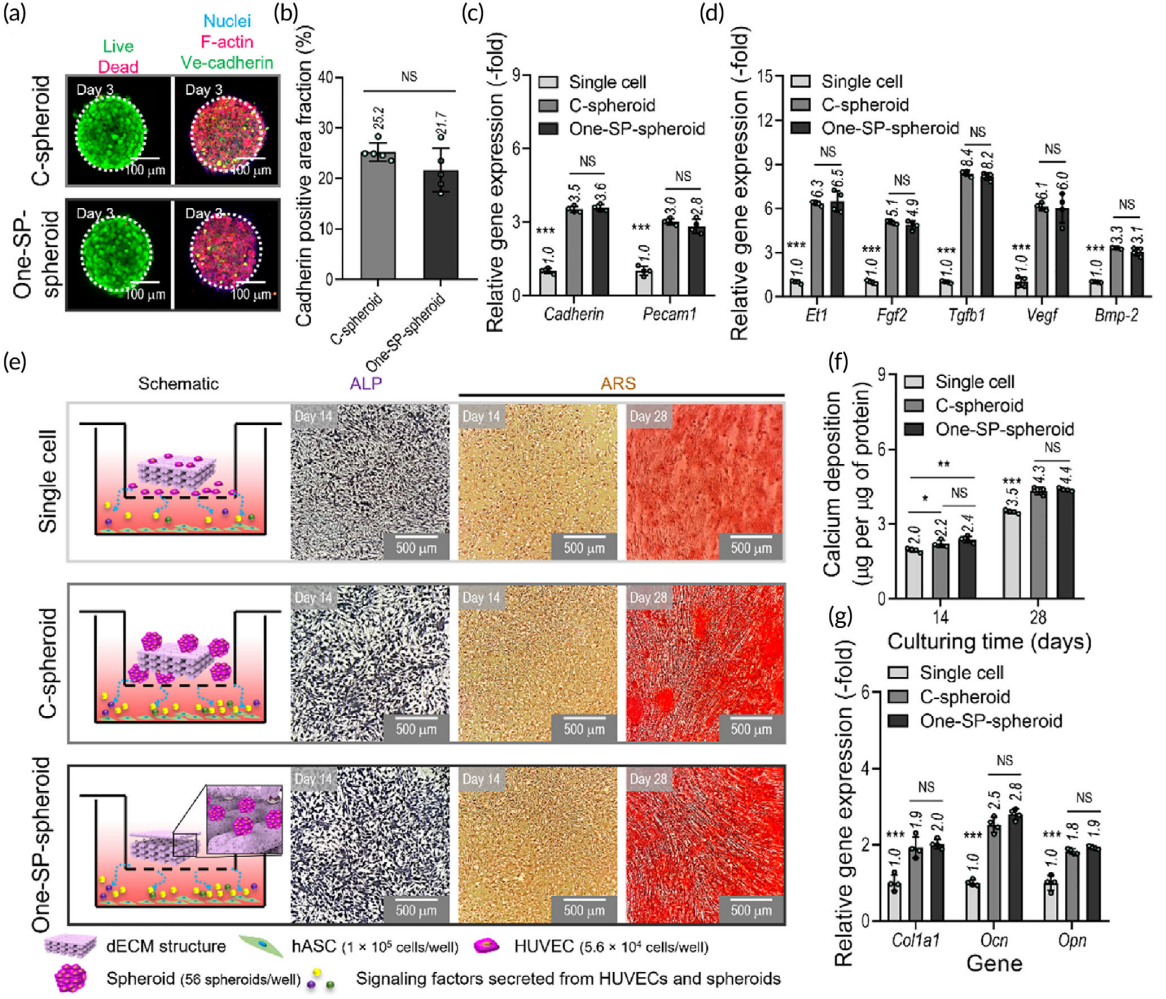
Comparison of in‐vitro cellular activities of One‐SP‐spheroids and conventional spheroids. (a) Live/dead and nuclei/F‐actin/VE‐cadherin images for conventional spheroids (C‐spheroid) and One‐SP‐spheroids at 3 days. (b) VE‐cadherin‐positive area fraction (%) of spheroids (*n* = 5). Expression of genes related to (c) endothelial cell–cell adhesion (*Ve‐cadherin* and *Pecam1*; at 3 days) and (d) signaling factors (*Et1*, *Fgf2*, *Tgfb1*, *Vegf*, and *Bmp‐2*; at 14 days) in the spheroid groups compared with single cells (*n* = 4). (e) Schematic diagrams illustrating the in‐vitro transwell model, in which hASCs (bottom chamber) and HUVECs (single cell, C‐spheroids, and One‐SP‐spheroids; upper chamber) were cocultured, ALP (14 days) staining, and ARS (14 and 28 days) staining of cultured hASCs. (f) Calcium deposition (at 14 and 28 days) and (g) expression of osteogenesis‐related genes (*Col1a1*, *Ocn*, and *Opn*; at 28 days) in cultured hASCs (*n* = 4). *p < 0.050, **p < 0.010, ***p < 0.001, Student's *t*‐test and one‐way ANOVA with Tukey's HSD post‐hoc test. ALP, alkaline phosphatase; ANOVA, analysis of variance; ARS, alizarin red S; hASCs, human adipose stem cells; HUVECs, human umbilical vein endothelial cells; One‐SP, one‐step spheroid printing; Tukey's HSD, Tukey's Honest Significant Difference

Live/dead and nuclei/F‐actin/Ve‐cadherin images of spheroids after 3 days in culture (Figure 5a) showed that preparation with either the One‐SP or conventional microwell methods yielded similar spheroid diameters (One‐SP‐spheroid: 206.1 ± 10.8 μm, C‐spheroid: 203.2 ± 5.3 μm) and areas (%) positive for VE‐cadherin (Figure 5b). To quantitatively evaluate cell–cell adhesion molecules required for stable spheroids, mRNA expression of *Ve‐cadherin* and *Pecam1* genes in the two types of spheroids was compared with that in 2D‐cultured single cells (Figure 5c). Expression of these genes was similar in the One‐SP‐spheroids and C‐spheroids and was significantly upregulated compared with that of single cells in 2D culture.

To observe signaling factors secreted by cells in the spheroids, measurements were made of the expression of various genes (endothelin1 [*Et1*], basic fibroblast growth factor [*Fgf2*], transforming growth factor beta 1 [*Tgfb1*], vascular endothelial growth factor [*Vegf*], and bone‐morphogenic protein 2 [*Bmp‐2*]) that activate osteogenesis and angiogenesis of stem cells (Figure 5d). Expression of these genes was similar in the two types of cell‐spheroids and significantly higher than in single cells in 2D culture.

In addition, to measure the ability of the cell‐spheroids to stimulate hASC osteodifferentiation, hASCs were cultured with one of the three cell constructs (single cells, C‐spheroids, or One‐SP‐spheroids) in the dECM mesh structure in transwell plates (Figure 5e). As shown in the ALP and ARS images (Figure 5e) and by measurements of calcium deposition (Figure 5f) at 14 and 28 days, coculture of the spheroids and hASCs significantly enhanced osteogenic activity of hASCs. In addition, expression of the osteogenic genes *Col1a1*, *Ocn*, and *Opn* at 28 days was significantly elevated in both spheroid groups compared with the single‐cell group (Figure 5g). These results are consistent with signaling between the HUVEC‐spheroids and hASCs having promoted robust hASC osteodifferentiation due to angiogenic and osteogenic growth factors secreted by each cell type.^40^, ^48^, ^49^, ^50^, ^51^

### 3.5 Use of One‐SP for regenerating bone tissue

As we developed the cell‐spheroid printing process, which can produce simultaneously cell‐spheroids and cell‐loaded constructs together, various applications of the system can be further extended in the several tissue engineering field. We carefully expect that the hybrid cell constructs, such as stem cell‐spheroid‐carrier, which can secret bioactive molecules (exosome, growth factor, cytokine, etc.),^18^ vascularized cell constructs for bone, muscle, liver, skin, etc.,^22^, ^40^, ^52^, ^53^, ^54^ and neuromuscular structure,^55^ can be obtained using the One‐SP process efficiently. In this study, we applied the One‐SP system to fabricate a vascularized cell construct for regenerating bone tissue.

**FIGURE 6:**
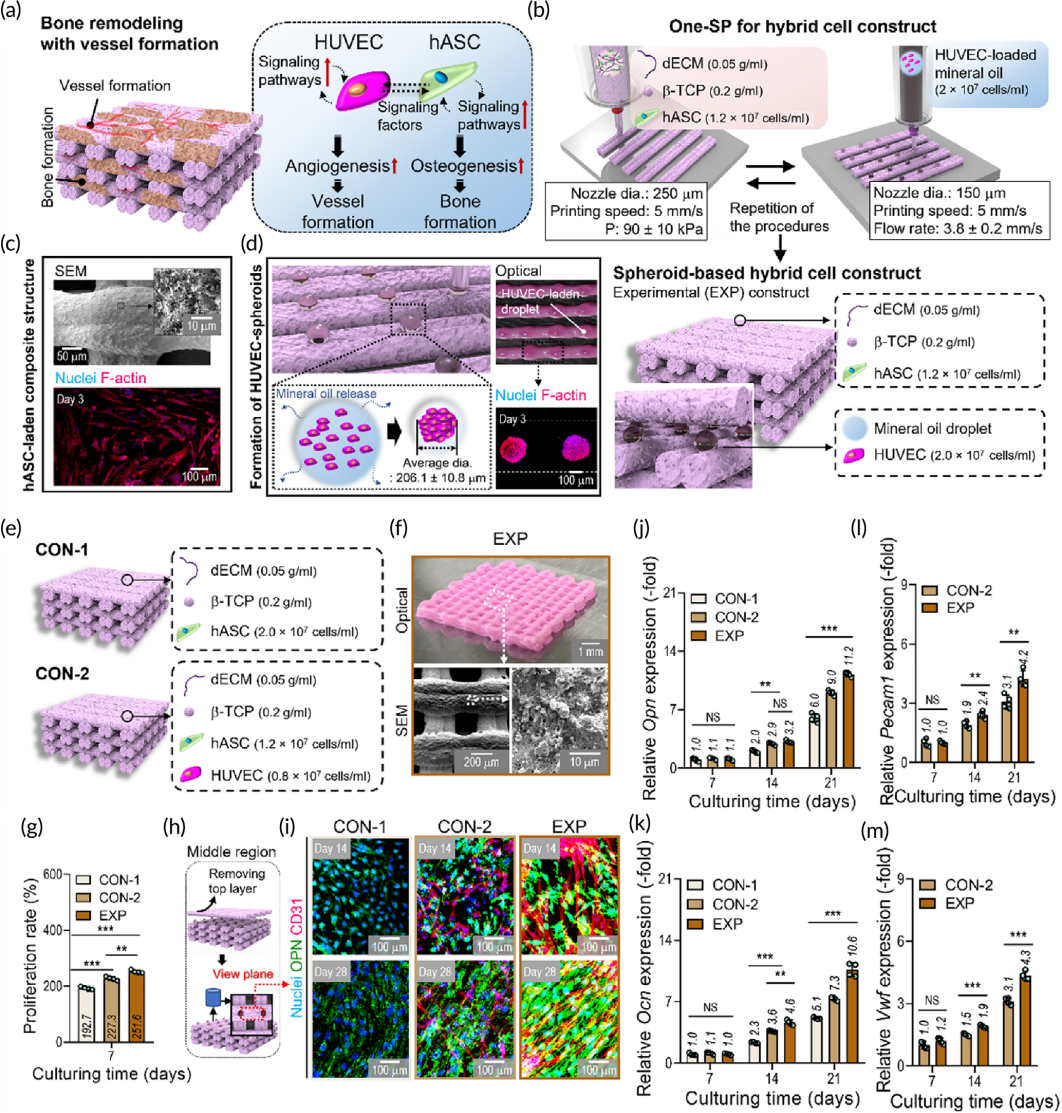
In‐vitro osteogenic activities of HUVEC‐spheroids in the hybrid cell constructs. (a) Schematic diagram of crosstalk between endothelial and stem cells to promote bone/vessel formation. (b) One‐SP to fabricate a spheroid‐based cell construct (EXP group) using hASC‐loaded dECM/β‐TCP bioink for struts and HUVEC‐loaded mineral oil droplets. (c) SEM and nuclei/F‐actin (at 3 days) images of a strut loaded with hASCs. (d) Schematic diagrams illustrating the spheroid‐forming procedure, and optical and nuclei/F‐Actin images of the fabricated HUVEC‐spheroids (at 3 days). (e) Schematics illustrating cell constructs fabricated using hASC‐loaded (CON‐1) and HUVEC/hASC‐loaded (CON‐2) dECM/β‐TCP bioinks, which were used as controls. (f) Optical and SEM images of the fabricated 3D EXP construct. (g) Proliferation rate of cells in the three cell constructs (CON‐1, CON‐2, and EXP), estimated with the MTT assay (*n* = 4). (h) Schematic diagram of the procedure for immunofluorescence imaging of spheroids within the constructs. (i) Nuclei/OPN/CD31 images of spheroids within constructs at 14 and 28 days. (J–M) Gene expression levels of (J) *Opn*, (K) *Ocn*, (L) *Pecam1*, and (M) *Vwf* (*n* = 4) for the constructs. **p < 0.010, ***p < 0.001, one‐way ANOVA with Tukey's HSD post‐hoc test and Student's *t*‐test. ANOVA, analysis of variance; dECM, decellularized extracellular matrix; hASC, human adipose stem cell; HUVEC, human umbilical vein endothelial cell; One‐SP, one‐step spheroid printing; SEM, scanning electron microscope; Tukey's HSD, Tukey's Honest Significant Difference

Several biofabrication methods, such as encapsulation of soluble factors and coculturing of multiple cells, have been employed to obtain efficient regeneration of bone tissues supported by effective blood vessel formation in vitro and in vivo.^56^ In particular, coculture of endothelial and stem cells has been widely used to promote formation of vascularized bone tissues in response to secretion of various signaling molecules that promote several signaling pathways involved in angiogenesis and osteogenesis of each cell type (Figure 6a). In this work, we identified synergistic effects between the endothelial and the stem cells that upregulate vessel and bone formation in vitro and compared them with pure hASC‐loaded bone constructs.

To improve upon previous results that took advantage of the synergism between HUVECs and hASCs, we used One‐SP to obtain a cell‐loaded construct that can more efficiently regenerate bone tissues. Figure 6b shows schematic diagrams of the One‐SP process. The supporting struts were fabricated using the hASC‐dECM/β‐TCP mixture (ratio = 2:8; cell density: 1.2 × 10^7^ cells/ml) derived from porcine bone, with the following printing parameters: nozzle diameter = 250 μm, pneumatic pressure = 90 ± 10 kPa, and printing nozzle speed = 5 mm/s. For depositing the HUVEC‐loaded droplets (cell density: 2 × 10^7^ cells/ml) onto the dECM/β‐TCP struts, the printing parameters were as follows: nozzle diameter = 150 μm, volume flow rate = 3.8 μl/min, printing nozzle speed = 5 mm/s (Figure 6b). As shown in the SEM and nuclei/F‐actin images in Figure 6c, the two parallel struts (diameter, about 150 μm) of the hASC‐loaded composite were used as a spheroid‐forming structure. The movement of the nozzle used to print the cell‐loaded mineral oil was perpendicular to the groove between the paired struts (Figure 6d). The procedures were repeated to obtain a 3D cell construct. The average diameter of the fabricated cell‐spheroids after 3 days in cell culture was about 206 μm, as shown in the nuclei/F‐actin image in Figure 6d. In addition, the fabricated hybrid cell construct was mechanically stable without collapsing after shaking it by forceps (Figure S5).

Two dECM/β‐TCP constructs were used as controls for evaluation of the in‐vitro cellular activities of the spheroid‐based cell constructs: hASC‐loaded (control‐1 [CON‐1]; 2.0 × 10^7^ cells/ml) and HUVEC/hASC‐loaded (control‐2 [CON‐2]; HUVECs, 0.8 × 10^7^ cells/ml; hASCs, 1.2 × 10^7^ cells/ml) (Figure 6e and Figure S6A). Optical and SEM images of the experimental group (EXP, the construct fabricated using One‐SP) are shown in Figure 6f, and those of the controls are shown in Figure S6B. Although the cell densities of the mineral oil bioinks used for the three constructs were different, the number of embedded cells and their ratios in the fabricated cell constructs (volume: 25 ± 1.2 mm^3^) were similar. CON‐1: hASC = 3.8 ± 0.2 × 10^5^ cells/sample; CON‐2: hASC = 2.4 ± 0.2 × 10^5^ and HUVEC = 1.6 ± 0.1 × 10^5^ cells/sample; EXP: hASC = 2.4 ± 0.1 × 10^5^ and HUVEC = 1.6 ± 0.1 × 10^5^ cells/sample. Detailed analyses of the cell constructs are shown in Supplementary Information (thermogravimetric analysis, Figure S6C, and compressive stress–strain curves, Figure S6D,E). As the compressive modulus of the EXP (26.3 ± 4.2 kPa, Figure S6E) was still low, it should be improved to apply the One‐SP to clinical applications in the future study.

To assess the proliferation rates of the cells at 7 days, MTT assays were performed on the experimental and two control groups (Figure 6g). Although cell proliferation of the constructs increased over the culture period, that for the CON‐2 (227.3%) and EXP (251.5%) groups was significantly greater than for the CON‐1 group (192.7%). This difference was the result of synergistic crosstalk between the endothelial and stem cells, which enhanced the intracellular activities of each,^48^, ^57^ and bioactive growth factors secreted from the HUVEC‐spheroids that accelerated cell.^51^, ^58^

To estimate osteogenic and angiogenic potential of the constructs, immunostaining and gene expression analyses were performed (Figure 6h–m and Figure S7). Nuclei/Opn (green)/CD31 (red) images for two regions ([1] top surface and [2] middle region after removal of the top surface) of the constructs were used to test for osteogenesis and angiogenesis. The EXP construct significantly enhanced osteogenic and angiogenic activities compared with those of the CON‐1 and CON‐2.

To quantitatively assess osteogenic and angiogenic differentiation of the cells, expression of genes related to early (*Alp* and *Runx2*)‐ and late (*Opn* and *Ocn*)‐stage osteogenic markers and endothelial markers (*Pecam1* and *Vwf*) were measured at 7, 14, and 21 days in culture (Figure 6j–m and Figure S7D). Expression of the early‐stage osteogenic genes *Alp* and *Runx2* was reduced at around 14 days in culture after reaching peak levels in all groups (Figure S7D). Furthermore, expression of the late osteogenic markers *Opn* and *Ocn* was significantly upregulated over time in culture and was highest in the EXP construct at 21 days (Figure 6j,k). In Figure 6l,m, expression of the endothelial markers *Pecam1* and *Vwf* was significantly higher in the EXP construct than in the CON‐2 construct. These results indicated that the EXP construct can efficiently promote secretion of paracrine factors and cellular interactions of hASCs and HUVEC‐spheroids, activating various signaling pathways and causing upregulation of cell proliferation and even osteogenic and angiogenic activities in the cells.

**FIGURE 7:**
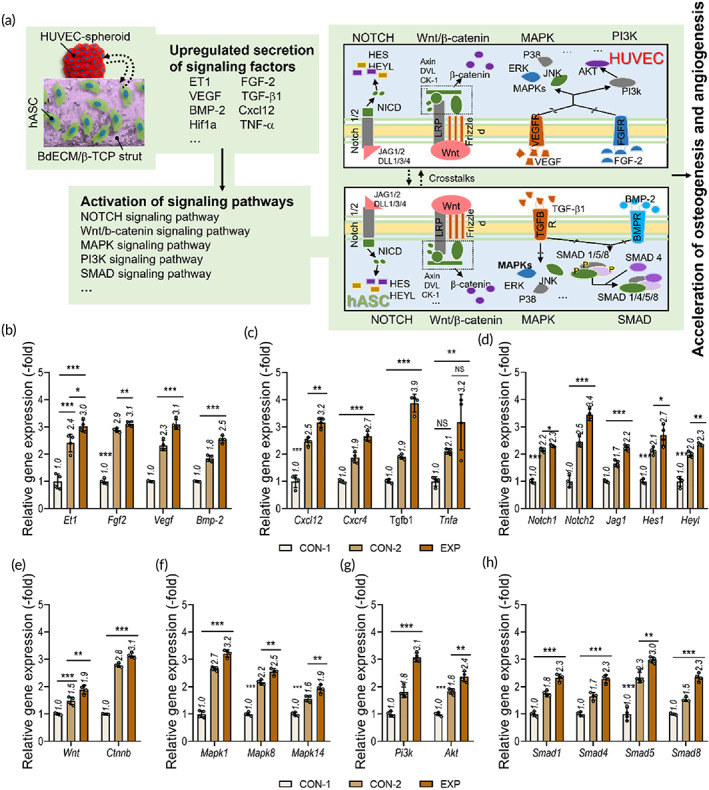
Expression of genes in hASCs and HUVEC‐spheroids in the cocultured hybrid constructs. (a) Schematic diagram illustrating the expected biological responses and crosstalk between cocultured hASCs and HUVEC‐spheroids in the EXP cell constructs. Expression of (b, c) signaling factors (*Et1*, *Fgf2*, *Vegf*, *Bmp*‐2, *Cxcl2*, *Cxcr4*, *Tgf‐β1*, *Tnfa*), (d) NOTCH (*Notch1*, *Notch2*, *Jag1*, *Hes1*, and *Heyl*), (e) Wnt/β‐catenin (*Wnt* and *Ctnnb*), (f) MAPK (*Mapk1*, *Mapk8*, and *MAPK14*), (g) PI3K (*Pi3k* and *Akt*), and (h) SMAD (*Smad1*, *Smad4*, *Smad5*, and *Smad8*) signaling pathway‐related genes in the CON‐1, CON‐2, and EXP constructs (*n* = 4). *p < 0.050, **p < 0.010, ***p < 0.001, one‐way ANOVA with Tukey's HSD post‐hoc test. ANOVA, analysis of variance; hASCs, human adipose stem cells; HUVECs, human umbilical vein endothelial cells; Tukey's HSD, Tukey's Honest Significant Difference

Expression of several signaling pathway genes was compared between the cell‐loaded constructs (CON‐1, CON‐2, and EXP). Figure 7a shows a schematic diagram of the upregulation of various signaling pathways related to osteodifferentiation in stem and endothelial cells by crosstalk between them.^40^, ^48^, ^49^, ^50^ To evaluate synergistic crosstalk within the constructs, expression of genes related to paracrine factors and several signaling pathways was measured (Figure 7b–h). Genes related to growth factors (*Et1*, *Fgf2*, *Tgfb1*, *Vegf*, and *Bmp‐2*), a cytokine (tumor necrosis factor alpha [*Tnfa*]), a chemokine (*Cxcl12*), and a chemokine receptor (*Cxcr4*) were activated to a greater extent in the EXP construct than in the controls. In addition, various signaling pathways, including the NOTCH (*Notch1*, *Notch2*, *Jag1*, *Hes1*, and *Heyl*), Wnt/β‐catenin (*Wnt* and *Ctnnb*), MAPK (*Mapk1*, *Mapk8*, and *Mapk14*), PI‐3K (*Pi3k* and *Akt*), and SMAD (*Smad1*, *Smad4*, *Smad5*, and *Smad8*) pathways, were also highly activated in the EXP construct due to enhanced secretion of signaling factors.

To confirm activation of signaling cascades and cellular responses in the two cell types, expression of representative genes from each pathway was estimated after 7, 14, and 21 days in culture (Figure S8). Activation of the NOTCH signaling pathway, which promotes cellular proliferation, was observed at early times, but was reduced after about 14 days in culture (Figure S8A).^59^ The Wnt/β‐catenin signaling pathway was significantly activated after 14 days in culture and affected early stages of osteogenesis and angiogenesis in hASCs and HUVEC‐spheroids (Figure S8B).^59^, ^60^ In addition, MAPK, PI‐3K, and SMAD signaling pathways were gradually activated over time in culture, inducing late stages of osteogenic differentiation and formation of vascular networks (Figure 7c–e).^59^, ^61^, ^62^, ^63^ Based on the gene expression analyses, we confirmed that the cellular activities of the spheroid‐based (EXP) construct could significantly promote osteogenic and angiogenic activities in these cells.

While the spheroid‐based cell constructs showed efficient osteogenic activities and vascular network formation in vitro, the mechanical properties of the construct were still low compared to those of natural bone. Considering the meaningful bioactive properties of the cell‐constructs, the structure could be applied into the non‐load bearing region of defected bones. However, the low mechanical properties should be handled in future to applicate the hybrid cell construct in various translational medicine fields.^64^ To improve the mechanical strength of the constructs, our previous strategies, such as printing core (high concentrated hydrogel)/shell (cell‐laden bioink) struts,^65^ poly(ε‐caprolactone)‐alginate interdigitated struts,^66^, ^67^, ^68^ and cell‐laden bioink coating on collagen/calcium deficient hydroxy apatite struts,^69^ can be applied.

Although the in vitro cellular activities demonstrated that the multiple‐cell‐constructs supported with the spheroids can provide a highly efficient platform to crosstalk between laden cells, the use of the mineral oil to fabricate the spheroids has several limitations to apply directly in clinical applications because the remnant mineral oil may cause inflammation and harmful effects in various tissues.^70^, ^71^ For this reason, the effect of the mineral oil that was used in the formation of the spheroids on in vivo results and complex biological functions will be studied in our future work. Additionally, more biocompatible and safer hydrophobic oils^72^, ^73^, ^74^ also could be considered for the One‐SP.

## 4 CONCLUSION

Here, we developed a novel 3D cell‐printing process in which cells aggregated into spheroids, and their location could be precisely manipulated to generate hybrid constructs containing multiple interacting cell types for use in regeneration of vascularized tissues. Toward that goal, a cell‐spheroid fabrication method was developed that used mineral oil loaded with cells, and spheroid formation was optimally selected by adjusting various 3D printing parameters. To demonstrate the feasibility of the in‐situ cell‐spheroid printing process, we used two cell types, endothelial cells and hASCs, and a cell‐supporting bioink consisting of a mixture of dECM derived from porcine bone and β‐TCP for use in regenerating vascularized bone tissue. The endothelial cells formed the cell‐spheroids, and homogeneously distributed hASCs were loaded into the struts of a printed 3D meshwork. The in‐situ spheroid‐based cell construct was characterized by several positive cellular responses, including increased cell proliferation and osteogenic and angiogenic activities, which were a result of significantly enhanced secretion of several growth factors and cytokines that contribute to synergistic intercellular crosstalk.

## AUTHOR CONTRIBUTIONS

**WonJin Kim:** Conceptualization (equal); formal analysis (equal); investigation (equal); methodology (equal); visualization (equal); writing – original draft (lead). **GeunHyung Kim:** Conceptualization (equal); formal analysis (equal); funding acquisition (lead); methodology (lead); project administration (lead); resources (lead); supervision (lead); visualization (equal); writing – review and editing (lead).

## CONFLICT OF INTEREST

The authors declare no conflict of interests.

### PEER REVIEW

The peer review history for this article is available at https://publons.com/publon/10.1002/btm2.10397.

## Supporting information


**Table S1** Primer sequences
**Figure S1**. Cell viability and spheroid sustainability cultured in GM with mineral oil
**Figure S2**. Mineral oil release after fabrication of hybrid cell constructs
**Figure S3**. Relationship between strut‐diameter and formed spheroid‐diameter
**Figure S4**. Preparation of cell‐spheroids by a conventional method
**Figure S5**. Structural stability of the hybrid structure
**Figure S6**. Characterization of fabricated bone constructs
**Figure S7**. In‐vitro osteogenic/angiogenic properties of the bioprinted constructs
**Figure S8**. Biological responses of hASCs and HUVEC‐spheroids in the fabricated construct

## Data Availability

The data has been included in the manuscript or Supplementary Information. Additional generated or analyzed data are available from the corresponding author upon reasonable request.
