# Supplementary material for: Hybrid cell constructs consisting of bioprinted cell‐spheroids
Source: Bioeng Transl Med. 2022 Aug 31;8(2):e10397. doi: 10.1002/btm2.10397 (PMC10013803; doi:10.1002/btm2.10397)
Supplement: Supplementary file 1 — Table S1 Primer sequences Figure S1. Cell viability and spheroid sustainability cultured in GM with mineral oil Figure S2. Mineral oil release after fabrication of hybrid cell constructs Figure S3. Relationship between strut‐diameter and formed spheroid‐diameter Figure S4. Preparation of cell‐spheroids by a conventional method Figure S5. Structural stability of the hybrid structure Figure S6. Characterization of fabricated bone constructs Figure S7. In‐vitro osteogenic/angiogenic properties of the bioprinted constructs Figure S8. Biological responses of hASCs and HUVEC‐spheroids in the fabricated construct [file BTM2-8-e10397-s001.docx]

Supplementary Information

**Hybrid cell constructs consisting of bioprinted cell-spheroids**

*WonJin Kim*,^1^ *GeunHyung Kim*^1,2,*^

^1^Department of Biomechatronic Engineering, College of Biotechnology and Bioengineering, Sungkyunkwan University (SKKU), Suwon 16419, South Korea

^2^Biomedical Institute for Convergence at SKKU (BICS), Sungkyunkwan University, Suwon 16419, South Korea

^*^Corresponding author at: Department of Biomechatronic Engineering, College of Biotechnology and Bioengineering, Sungkyunkwan University (SKKU), Suwon 16419, South Korea. E-mail address: gkimbme@skku.edu (G.H. Kim).

**Supplementary Information contains:**

Supplementary Table

Table S1. Primer sequences

Supporting Figures

Figure S1. Cell viability and spheroid sustainability cultured in GM with mineral oil

Figure S2. Mineral oil release after fabrication of hybrid cell constructs

Figure S3. Relationship between strut-diameter and formed spheroid-diameter

Figure S4. Preparation of cell-spheroids by a conventional method

Figure S5. Structural stability of the hybrid structure

Figure S6. Characterization of fabricated bone constructs

Figure S7. *In-vitro* osteogenic/angiogenic properties of the bioprinted constructs

Figure S8. Biological responses of hASCs and HUVEC-spheroids in the fabricated construct

**Table**

**Table S1. Primer sequences.** Information of applicated primers for qRT-PCR analysis.

| Gene | Source | Primer sequence | | GeneBank number |
| --- | --- | --- | --- | --- |
|  |  | Left (5’ – 3’) | Right (5’ – 3’) |  |
| *Gapdh* | *Homo sapiens* | CCATGGGGAAGGTGAAGGTC | AGTGATGGCATGGACTGT | NM_002046.7 |
| *Ve-cadherin* | *Homo sapiens* | CTTCACCCAGACCAAGTACACA | TGTTGGCCGTGTTATCGTGA | NM_001795.5 |
| *Pecam1* | *Homo sapiens* | TGAGTGGTGGGCTCAGATTG | TGAGTCTAGGTCGGGGAGTG | NM_000442.5 |
| *Col1a1* | *Homo sapiens* | TGACGAGACCAAGAACTGCC | GCACCATCATTTCCACGAGC | NM_000088.4 |
| *Et1* | *Homo sapiens* | CTGCCTTTTCTCCCCGTTAAA | AGCCAGTGAAGATGGTTGGG | NM_001168319.2 |
| *Fgf2* | *Homo sapiens* | CCCAGAAAACCCGAGCGA | AGGAAGAAGCCCCCGTTTTT | NM_002006.5 |
| *Tgf-β1* | *Homo sapiens* | GGGCTACCATGCCAACTTCT | GCACTTCAACAGTGCCCAAG | NM_000660.7 |
| *Vegf* | *Homo sapiens* | AGGCCAGCACATAGGAGAGA | ACGCGAGTCTGTGTTTTTGC | NM_001171623.1 |
| *Bmp-2* | *Homo sapiens* | CAGACCACCGGTTGGAGA | CCACTCGTTTCTGGTAGTTCTTC | NM_001200.4 |
| *Cxcl12* | *Homo sapiens* | CGATTCTTCGAAAGCCATTATTGT | GATCAGTGGGGGAACCAAGG | NM_ 001277990.2 |
| *Cxcr4* | *Homo sapiens* | AGGGGATCAGTATATACACTTCAGA | GAGGATCTTGAGGCTGGACC | NM_003467.3 |
| *Tnfα* | *Homo sapiens* | CTGGGCAGGTCTACTTTGGG | CTGGAGGCCCCAGTTTGAAT | NM_000594.4 |
| *Notch1* | *Homo sapiens* | CCAGCATCACCTGCCTGTTA | CCAAGTCTGACGTCCCTCAC | NM_000214.3 |
| *Notch2* | *Homo sapiens* | CTACAGTTGTCGCTGCTTGC | GTTGGAGAGGCACTCGTTGA | NM_024408.4 |
| *Jag1* | *Homo sapiens* | GGCCGAGGTCCTATACGTTG | ACACAAGGTTTGGCCTCACA | NM_000214.3 |
| *Hes1* | *Homo sapiens* | ATGACAGTGAAGCACCTCCG | AAACACCTTAGCCGCCTCTC | NM_005524.4 |
| *Heyl* | *Homo sapiens* | CGCCATGAAGCGACCCAAG | GTAAGCAGCCGACCCTGTAG | NM_014571.4 |
| *Wnt* | *Homo sapiens* | CGCCATGAAGCGACCCAAG | GTAAGCAGCCGACCCTGTAG | NM_005430.4 |
| *Ctnnb* | *Homo sapiens* | CAAGATCGTCAACCGAGGCT | AAGGTTCATGAGGAAGCGCA | NM_001098209.2 |
| *Mapk1* | *Homo sapiens* | AGTTCTTGACCCCTGGTCCT | CCTGGGACATCCCCAGAAAC | NM_002745.5 |
| *Mapk8* | *Homo sapiens* | CTTGGCATGGGCTACAAGGA | GCCATTGATCACTGCTGCAC | NM_139049.4 |
| *Mapk14* | *Homo sapiens* | CCAGGGGCTGAGCTTTTGAA | AGCAACCAGAAGGTATGGGC | NM_139012.3 |
| *Pi3k* | *Homo sapiens* | TCAGCAGGCAAAGACCGATT | GCTGACCATGCTGCTATGAAC | NM_006218.4 |
| *Akt* | *Homo sapiens* | CCAGGATCCATGGGTAGGAAC | CTCCTCCTCCTCCTGCTTCT | NM_001382430.1 |
| *Smad1* | *Homo sapiens* | CAATCCAGGCTCCAGGAGAAAG | GAAAAGTGGCGTTGAGTGGC | NM_005900.3 |
| *Smad4* | *Homo sapiens* | CCATCCAGCATCCACCAAGT | TGTCGATGACACTGACGCAA | NM_005359.6 |
| *Smad5* | *Homo sapiens* | TAGCCGGCTCGCGAAAAG | GGGTCAAGTCAGAGGCAGATTT | NM_005903.7 |
| *Smad8* | *Homo sapiens* | ACCCCTGCCTTATCATGCCA | TCAGCACCCCAACCCTTAAC | NM_001127217.3 |
| *Alp* | *Homo sapiens* | GGCACCTGCCTTACTAACTCC | CTTGCCACGTTGGTGTTGA | NM_000478.6 |
| *Runx2* | *Homo sapiens* | CAGTGACACCATGTCAGCAA | GCTCACGTCGCTCATTTTG | NM_001024630.4 |
| *Opn* | *Homo sapiens* | AAGTTTCGCAGACCTGACATC | GGGCTGTCCCAATCAGAAGG | NM_000582.2 |
| *Ocn* | *Homo sapiens* | TGAGAGCCCTCACACTCCTC | ACCTTTGCTGGACTCTGCAC | NM_199173.6 |
| *Vwf* | *Homo sapiens* | ACACCTGCATTTGCCGAAAC | ATGCGGAGGTCACCTTTCAG | NM_000552.5 |

Gapdh: glyceraldehyde-3-phosphate dehydrogenase; *Pecam1*: platelet and endothelial cell adhesion molecule 1 (CD31); *Col1a1*: Collagen type I alpha 1 chain; *Et1*: endothelin 1; *Fgf2*: fibroblast growth factor 2; *Tgf-β1*: transforming growth factor beta 1; *Vegf*: vascular endothelial growth factor; *Bmp-2*: bone-morphogenic protein 2; *Cxcl12*: C-X-C motif chemokine ligand 12 (SDF-1); *Cxcr4*: C-X-C motif chemokine receptor 4; *Tnf*α: tumor necrosis factor alpha; *Jag1*: jagged canonical NOTCH ligand 1; *Hes1*: hes family bHLH transcription factor 1; *Heyl*: hes-related family bHLH transcription factor with YRPW motif like; *Ctnnb*: beta catenin; *Mapk1*: mitogen-activated protein kinase 8 (Erk); *Mapk8*: mitogen-activated protein kinase 8 (Jnk); *Mapk148*: mitogen-activated protein kinase 148 (P38); *Pi3k*: phosphoinositide 3-kinase; *Akt*: Protein kinase B (PKB) ; *Alp*: alkaline phosphatase; *Runx2*: RUNX family transcription factor 2; *Opn*: bone sialoprotein I (*Spp1*); *Ocn*: bone gamma-carboxyglutamate protein (*Bglap*); *Vwf*: von Willebrand factor.

**Figures**


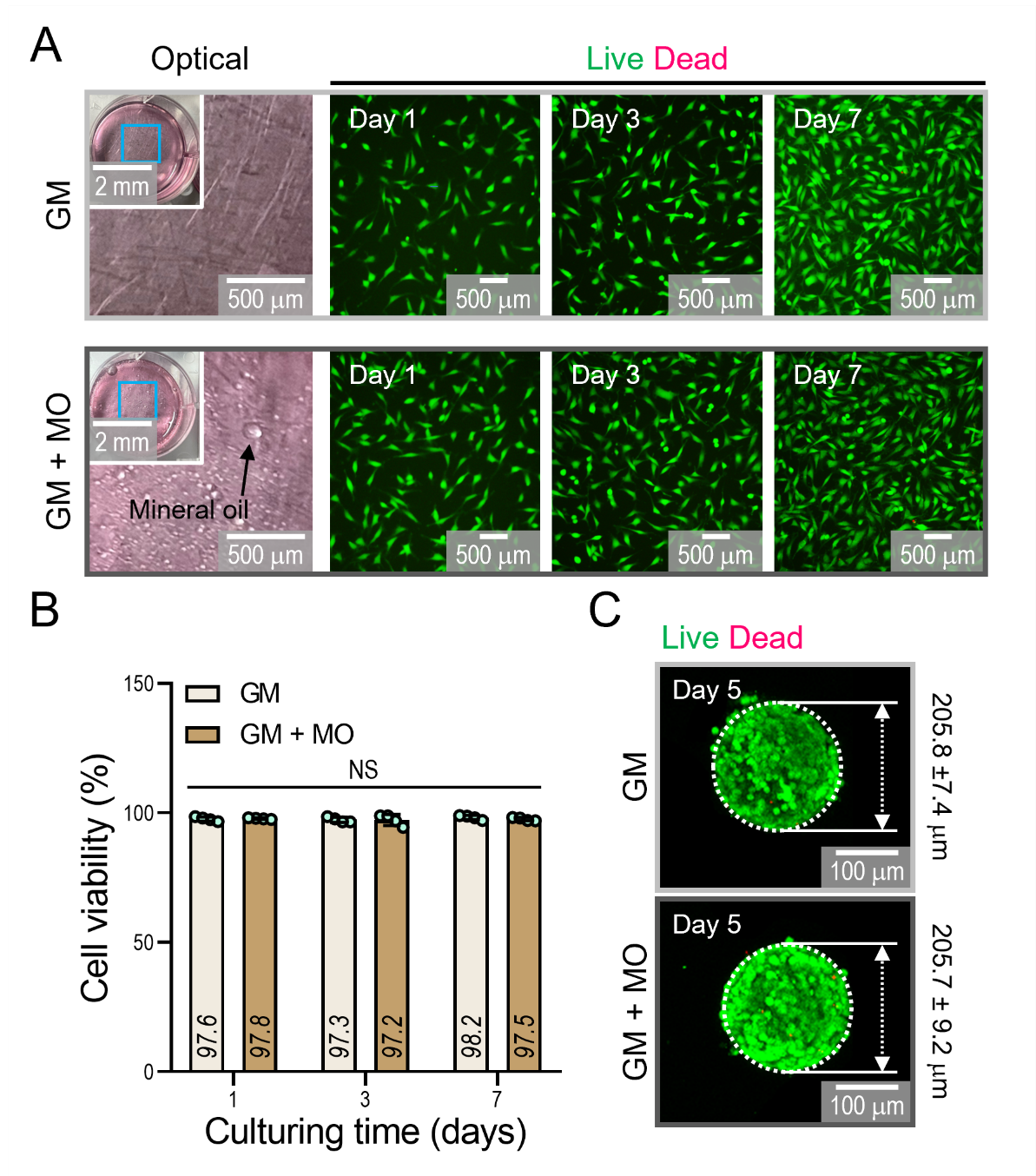


**Figure S1. Cell viability and spheroid** **sustainability cultured in GM with mineral oil.** (A) Optical and live/dead (at 1, 3, and 7 d of culture) of HUVECs (cell density: 5 × 10^4^ cells/well) cultured with GM only and GM with mineral oil (MO; 10 v/v%). (B) Cell viability of the cultured HUVECs (n = 4). (C) Live/dead images of the One-SP-spheroids cultured with GM only and GM with MO.


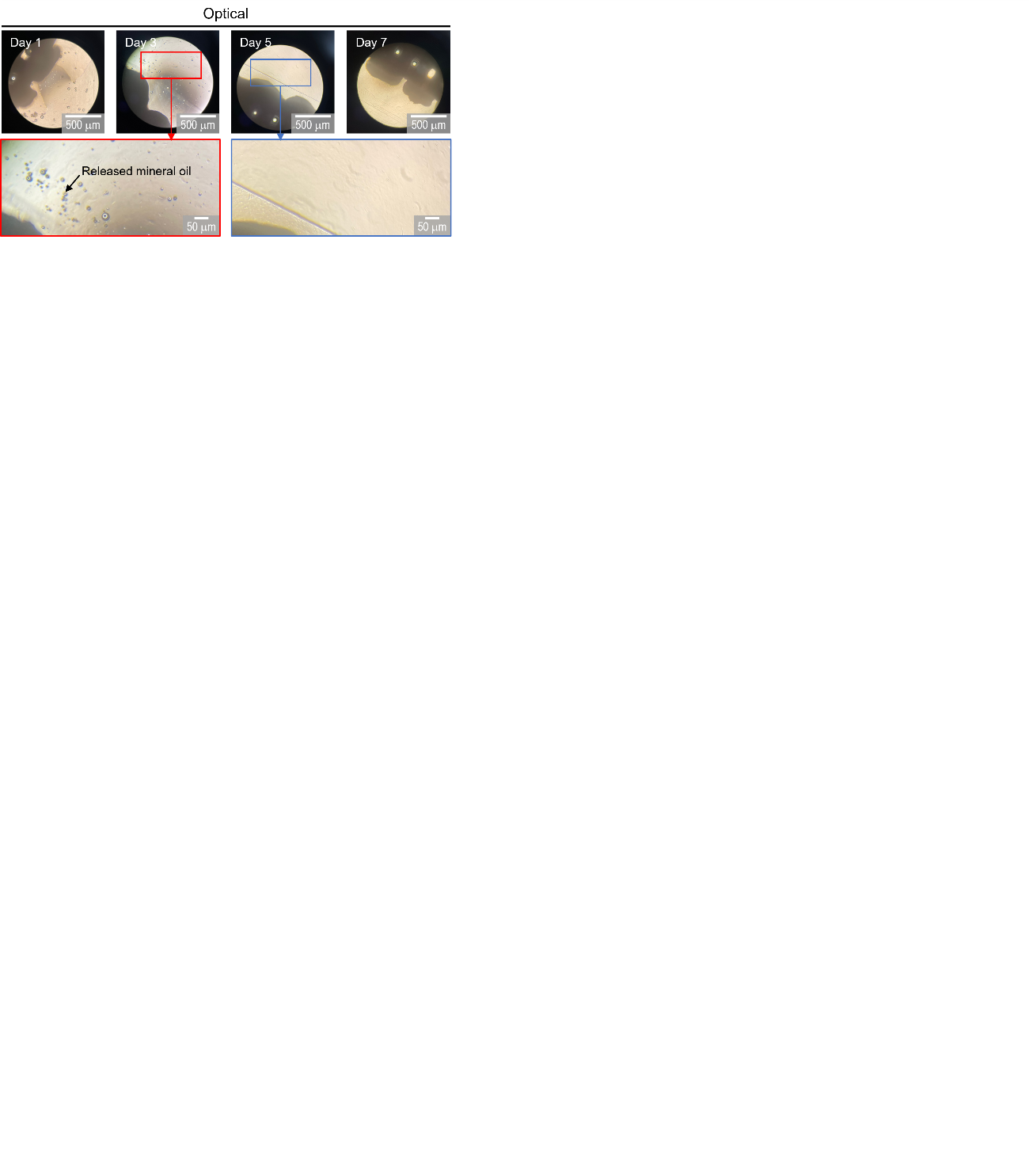


**Figure S2. Mineral oil release after fabrication of hybrid cell constructs.** Optical images showing the release of mineral oil from the fabricated hybrid cell constructs at 1, 3, 5, and 7 d of culture in the GM.


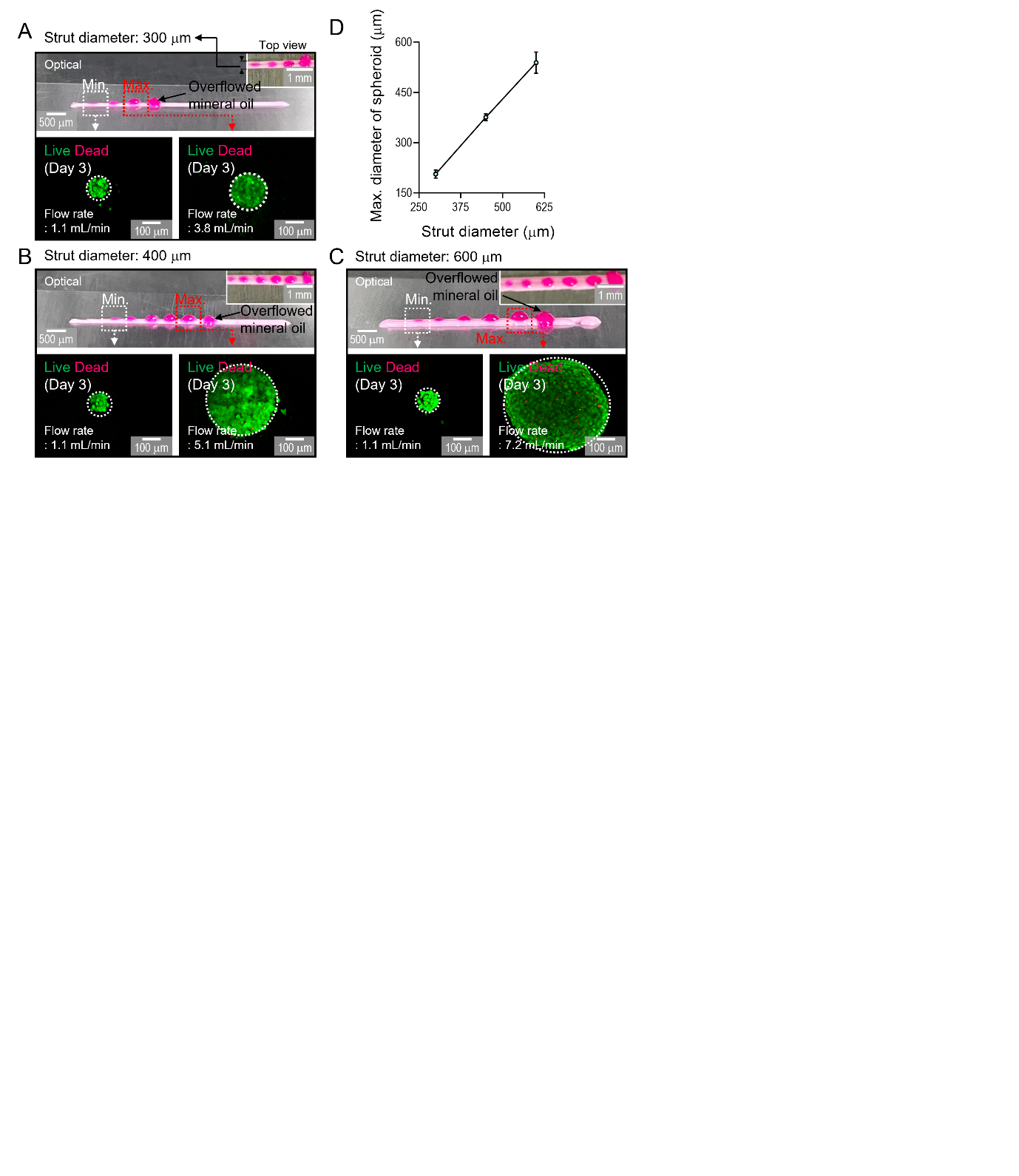


**Figure S3.** **Relationship between strut-diameter and formed spheroid-diameter.** Optical and live/dead images showing various deposited mineral oil droplets and fabricated HUVEC-spheroids onto the three different strut-diameters [(A) 300, (B) 400, and (C) 600 μm]. (D) Maximum spheroid-diameters for the struts (n = 10).


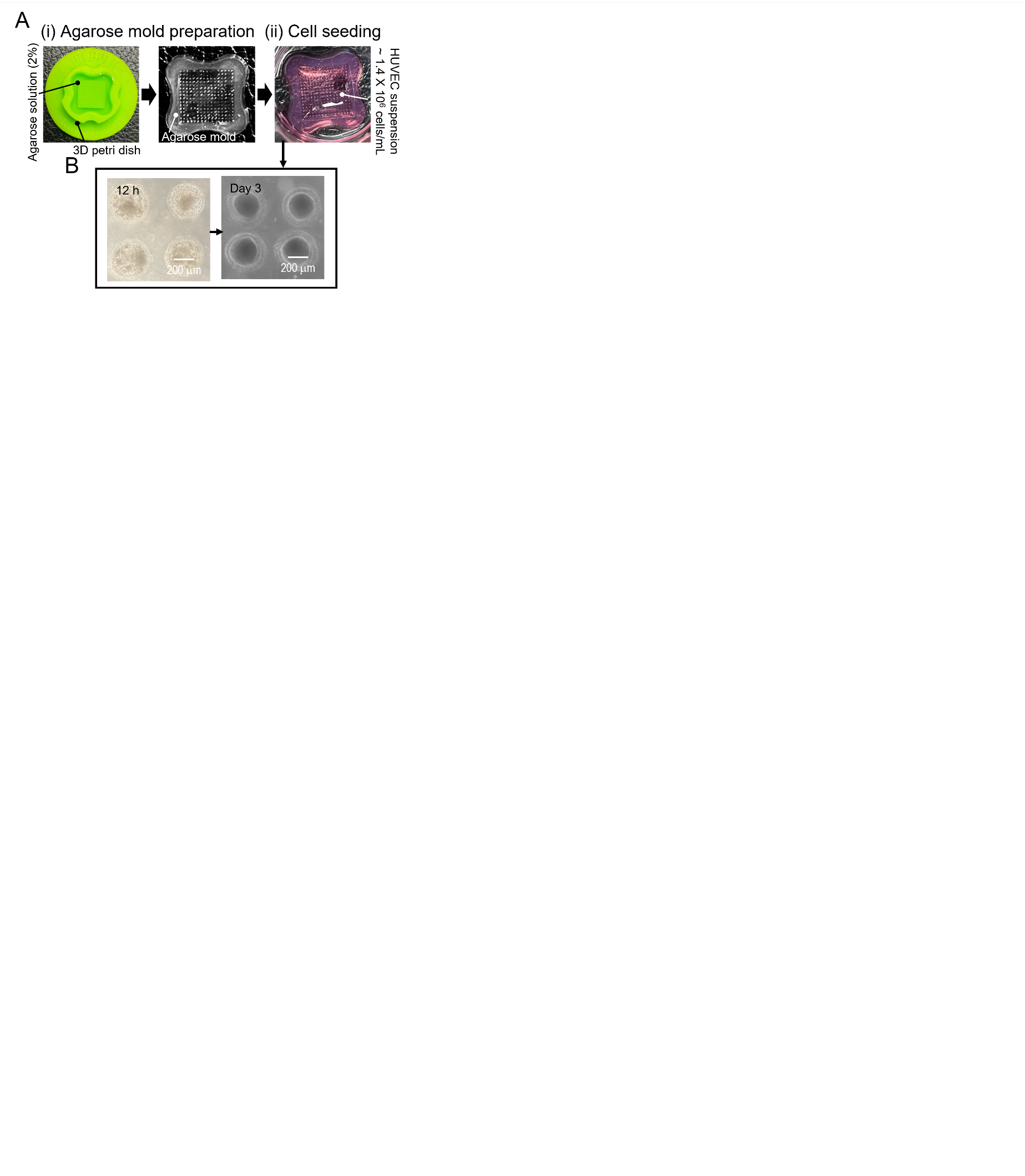


**Figure S4.** **Preparation of cell-spheroids by a conventional method.** (A) Optical images showing the procedures of preparing C-spheroids; i) preparing agarose mold and ii) seeding HUVEC suspension (190 μL per mold) into the agarose mold. (B) Optical images at 12 h and 3 d of culture for the HUVECs seeded in the agarose mold showing formation of C-spheroids.

**
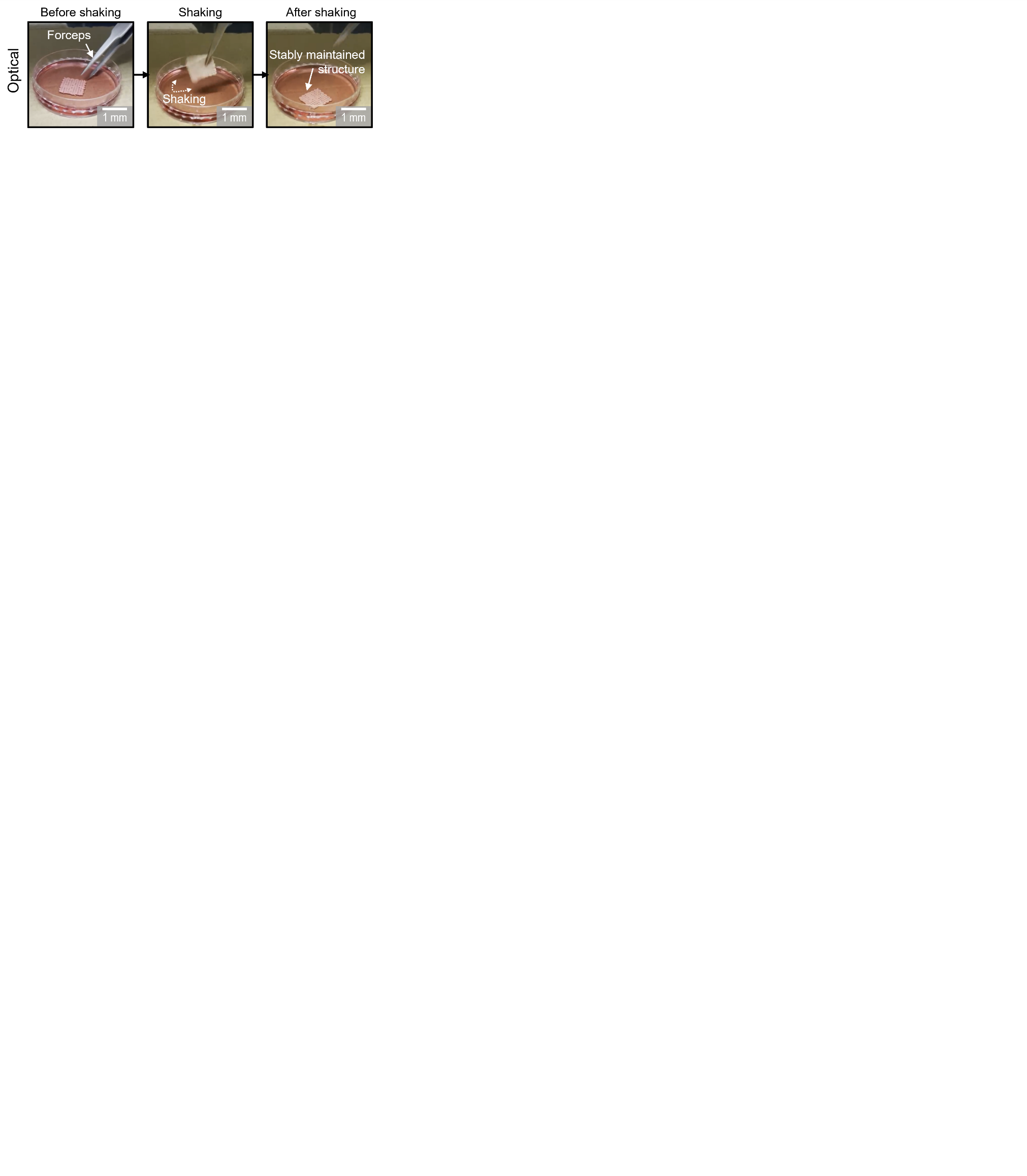
**

**Figure S5.** **Structural stability of a hybrid structure.** Optical images of the printed hybrid construct before and after shaking with forceps.


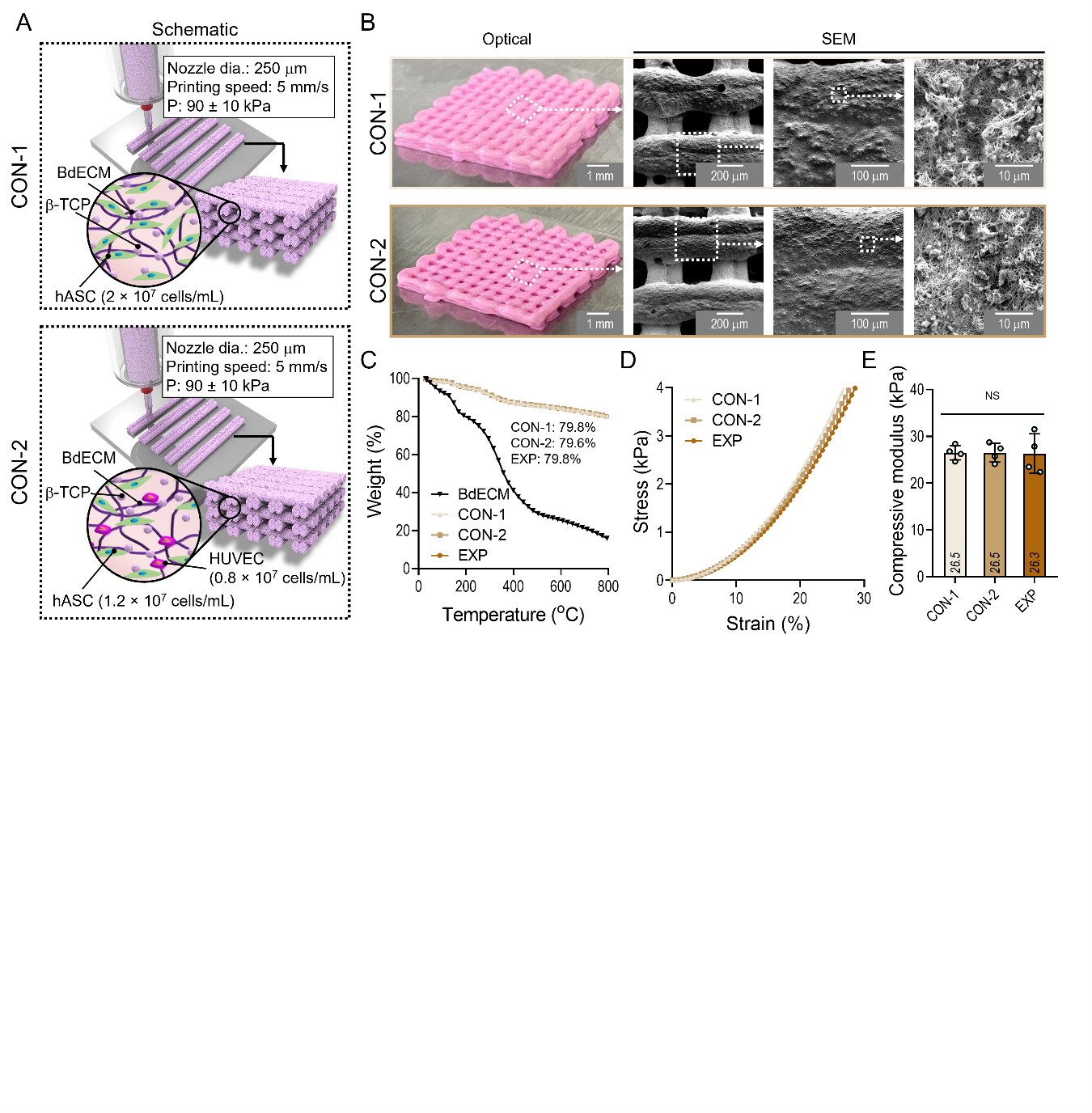


**Figure S6.** **Characterization of fabricated bone constructs.** (A) Illustrated schematics demonstrating fabrication of hASC-loaded dECM/β-TCP (CON-1) and HUVEC/hASC-loaded dECM/β-TCP (CON-2) constructs. (B) Optical and SEM images of the CON-1 and CON-2 constructs. (C) Thermogravimetric analysis of the bioprinted scaffolds and the pure dECM powder. (D) Stress-strain curves and (E) compressive moduli of the scaffolds. One-way ANOVA and Tukey’s HSD post-hoc test were used to test for significance.


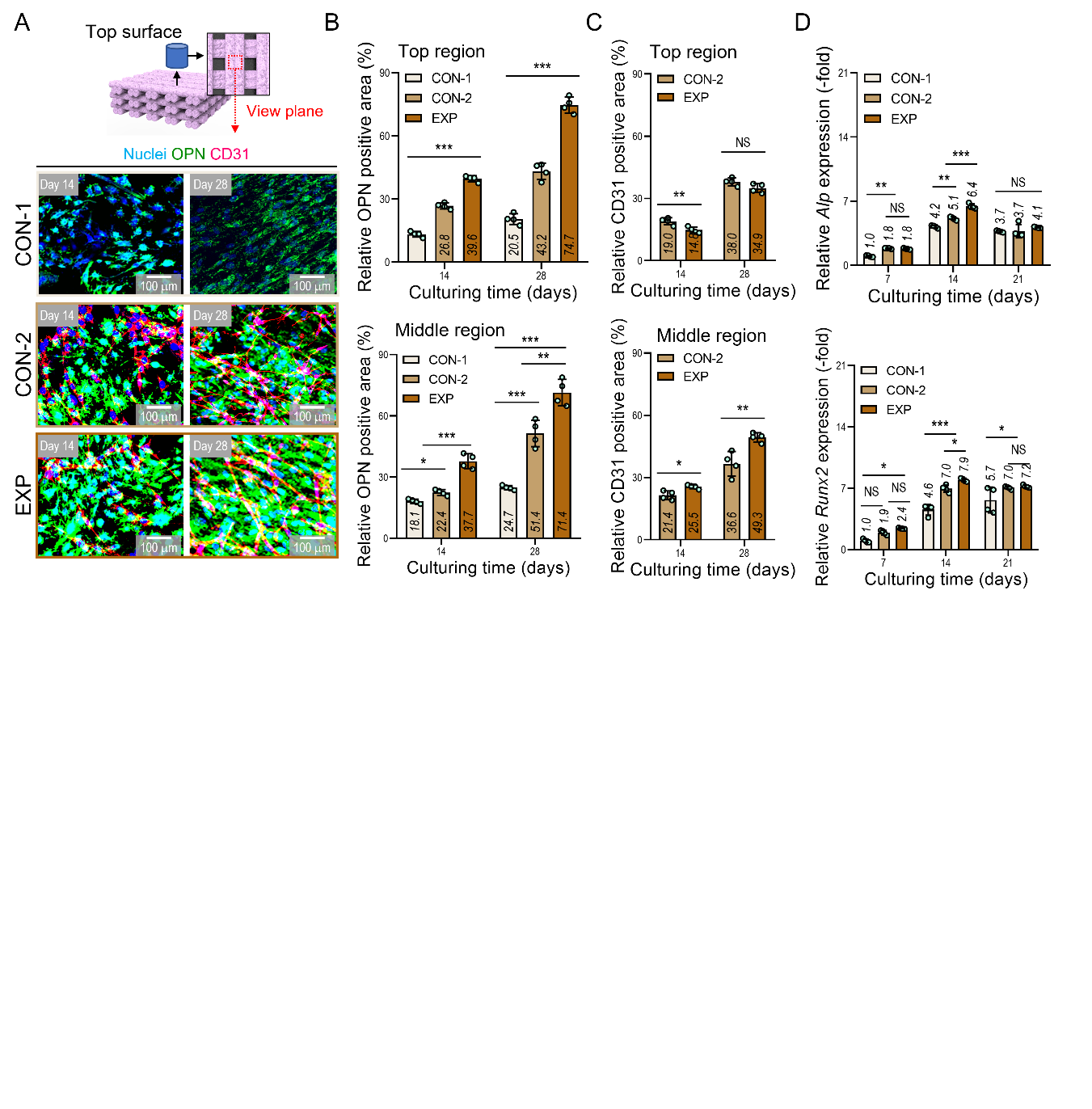


**Figure S7.** ***In-vitro* osteogenic/angiogenic properties of the bioprinted constructs.** (A) Nuclei/OPN/CD31 images observed at the top surface of the CON-1, CON-2, and EXP constructs at 14 and 28 d. Quantified (B) OPN and (C) CD31-positive areas estimated from the immunofluorescent images at the top and middle regions (n = 4). (D) Expression levels of osteogenesis-related genes, including *Alp* and *Runx2* (n = 4). ^*^*p* < 0.050, ^**^*p* < 0.010, ^***^*p* < 0.001, one-way ANOVA with Tukey’s HSD post-hoc test and Student’s *t*-test.


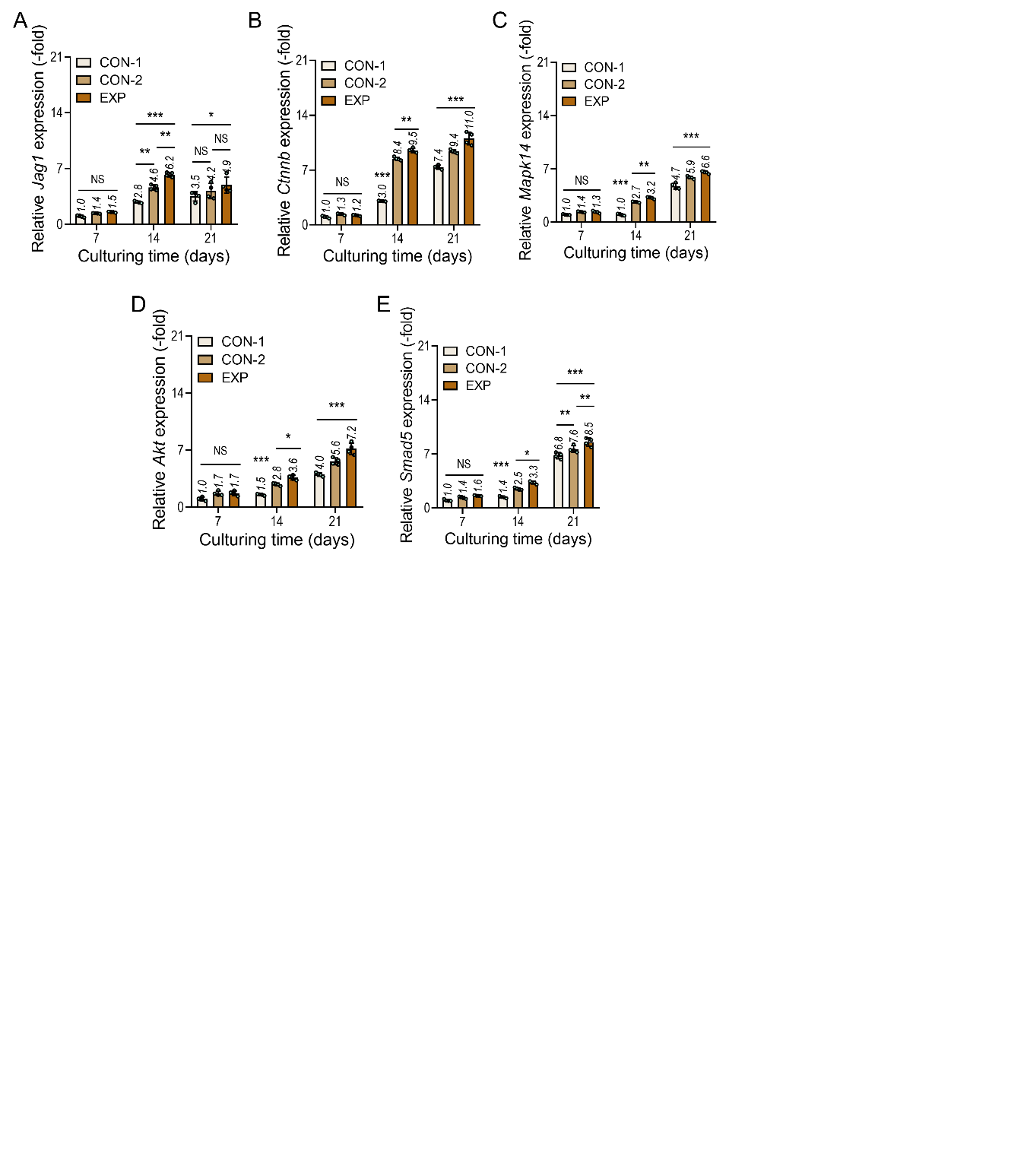


**Figure S8.** **Biological responses of hASCs and HUVEC-spheroids in the fabricated construct.** (A) NOTCH (*Jag1*), (B) Wnt/β-catenin (*Ctnnb*), (C) MAPK (*Mapk14*), (D) PI3K (*Akt*), and SMAD (*Smad5*) signaling pathway-related gene expression in the CON-1, CON-2, and EXP constructs at 7, 14, and 21 d (n = 4). ^*^*p* < 0.050, ^**^*p* < 0.010, ^***^*p* < 0.001, one-way ANOVA with Tukey’s HSD post-hoc test.
